# *Actinidia* DRM1 - An Intrinsically Disordered Protein Whose mRNA Expression Is Inversely Correlated with Spring Budbreak in Kiwifruit

**DOI:** 10.1371/journal.pone.0057354

**Published:** 2013-03-13

**Authors:** Marion Wood, Georgina M. Rae, Rong-Mei Wu, Eric F. Walton, Bin Xue, Roger P. Hellens, Vladimir N. Uversky

**Affiliations:** 1 Genomics Research, The New Zealand Institute for Plant & Food Research Limited, Auckland, New Zealand; 2 Research for Enterprise, University of Otago, Auckland, New Zealand; 3 Department of Molecular Medicine, University of South Florida, Tampa, Florida, United States of America; 4 Institute for Biological Instrumentation, Russian Academy of Sciences, Pushchino, Moscow Region, Russia; Wuhan University, China

## Abstract

Intrinsically disordered proteins (IDPs) are a relatively recently defined class of proteins which, under native conditions, lack a unique tertiary structure whilst maintaining essential biological functions. Functional classification of IDPs have implicated such proteins as being involved in various physiological processes including transcription and translation regulation, signal transduction and protein modification. *Actinidia* DRM1 (Ade *DORMANCY ASSOCIATED GENE 1*), represents a robust dormancy marker whose mRNA transcript expression exhibits a strong inverse correlation with the onset of growth following periods of physiological dormancy. Bioinformatic analyses suggest that DRM1 is plant specific and highly conserved at both the nucleotide and protein levels. It is predicted to be an intrinsically disordered protein with two distinct highly conserved domains. Several *Actinidia* DRM1 homologues, which align into two distinct *Actinidia*-specific families, Type I and Type II, have been identified. No candidates for the *Arabidopsis* DRM1-Homologue (AtDRM2) an additional family member, has been identified in *Actinidia.*

## Introduction

Perception of decreasing temperature and day length by perennial species in late summer is associated with the termination of growth, cold acclimation, transition to endodormancy and culmination in maximal cold hardiness [1]. Developmentally, buds are initiated and mature beneath bud scales which are designed to protect the delicate meristems within [2], [3]. Earlier initiated axillary buds are thought to transition from paradormancy through to endodormancy, in late autumn/early winter, via a complex signalling cascade that includes both exogenous and endogenous cues [4], [5]. With the perception of increasing daily temperatures endodormant buds undergo a transition to ecodormancy. In the case of kiwifruit (*Actinidia deliciosa* (Ade) ‘Hayward’), buds display an increase in sap flow and respiration three to eight weeks prior to budburst [6]. It is postulated that this transition from endodormancy to ecodormancy primes the plant for a rapid response upon perception of favourable growth conditions [7]. In temperate perennial species, such as kiwifruit, a sustained period of winter chilling, which is accumulated during exposure to low temperatures, is required to optimise bud release from endodormancy [8], [9].

In warmer regions, an application of hydrogen cyanamide (hereafter referred to as HC) in late winter/early spring is often used to optimise budburst in kiwifruit vines and to ensure commercially viable yields are achieved [10]. An understanding of how the release from dormancy is regulated, at both the molecular and physiological levels, is essential in order to manipulate temperate crops successfully. Many reviews have focused on the general physiological aspects of bud dormancy [7], [11] and a number of recent studies have focused on the molecular aspects of HC-induced budbreak in grapes [12], [13]. However, the precise mode of action of HC remains unclear.

One gene family that has long been associated with dormancy and is routinely used as a marker for paradormancy release is DORMANCY 1 (DRM1)/Auxin Repressed Protein (ARP) gene family (DRM1/ARP) [14], [15]. Two family members in *Arabidopsis thaliana* are highly conserved at the protein level: namely Ath_DRM1 (NP_001154378) and Ath_DRM2 (NP_850220). Expression profiling of Ath_DRM1 and Ath_DRM2 suggests a strong inverse correlation with the growth potential of the plant, and this relationship has also been observed in other plant species [14], [15], [16], [17], [18]. Generally, the deduced DRM1 protein is in the order of 92–155 amino acids, with a predicted molecular mass of between 11 and 14 KDa. No family members exhibit signal peptide or organellar targeting sequences, but the tagged protein product has been shown to be expressed in the cytosol of *Arabidopsis* protoplasts [19]. Previous multiple sequence alignment of the DRM1 gene family indicates the presence of two distinct clades namely Clade 1 and Clade 2, characterised by the presence of either two conserved domains (Domain 1 and 2) or Domain 1 only, respectively [18], [19]. Recently, Finlayson [20] demonstrated that the Arabidopsis *tbl1* null mutants exhibited a hyper-branching, non-pleiotrophic phenotype in a DRM1-independent manner. These data suggest that dormancy release and bud outgrowth may be regulated through several independent response pathways analogous to what is observed with the flowering response.

This research was undertaken to gain a better understanding of the role of DRM1/ARP family in kiwifruit bud dormancy. An inverse correlation between Ade_DRM1 transcript expression and the growth potential of the kiwifruit bud is documented and indeed, it is likely that the DRM1 gene family represents a plant-specific stress-related protein family. The Ade_DRM1 gene and associated homologues in other plant species have been bioinformatically predicted to contain regions of intrinsic disorder, which have been shown to be associated with regulatory functions such as transcription or signal transduction [21]. Multiple sequence alignment with an extended dataset has highlighted an expansion of Clade 2 characterised by the partial conservation of the second domain, building upon the observations of Steiner et al. [18] and Kim et al. [19]. No apparent *Actinidia* homologues have been identified for Ath_DRM2. Intra-*Actinidia* DRM1 sequence analyses have revealed two distinct families; Type I and Type II, which both exhibit high sequence identity with Ath_DRM1.

## Materials and Methods

### Plant Material and Sample Collection

Experiments were performed using kiwifruit (*Actinidia deliciosa* (A. Chev.) C.F. Liang et A.R. Ferguson ‘Hayward’) vines growing in commercial orchards in Hamilton (2000) and Kerikeri (2004 and 2005), New Zealand. No specific permits were required for the described field studies and full permission was granted by Plant and Food Research, NZ. Kiwifruit vine management was undertaken using standard orchard practices.

#### A: Winter series - 2005

In order to collect a population of buds where subsequent budbreak could be expected to be similar, only upward facing buds (excluding the most distal bud) were collected from one-year-old canes (mature dormant shoots) and immediately frozen in liquid nitrogen [22]. HC was applied to the vines in late winter (23 August 2005) at a rate of 6%, 600 L/ha^−1^ (as Hi-Cane®, NuFarm, New Zealand; active ingredient HC 520 g L^−1^), before any growth could be observed. Buds were collected 105, 90, 75, 51, 33, 18 and 1 days before HC application and 1, 2 and 3 days after HC application from both treated and non-treated vines.

#### B: Budbreak - 2000

Buds and resultant shoots were collected as described above except that no allowance was made for bud orientation. HC application was on 8 August 2000 at a rate of 6%, 600 L/ha^−1^ (as Hi-Cane, NuFarm, New Zealand; active ingredient HC 520 g L^−1^), and harvest commenced the day before HC application and then 6, 20, 34, 48, 62, 77 and 90 days after HC application from both treated and non-treated vines.

#### C: Budbreak - 2004

From each non-HC treated kiwifruit cane, two separate populations of whole buds were collected i) the most distal upward facing buds (Distal buds) and ii) the subsequent upward facing buds as one moved basally down the cane (Basal buds). *Note*: due to the phyllotaxis of kiwifruit shoots, the ‘Basal buds’ were generally the four buds back from the ‘Distal buds’. Harvest commenced on 24 August 2004 (one day prior to the application of HC to kiwifruit vines elsewhere in the orchard). Subsequent samples were collected 6, 13, 20, 27, 34 and 41 days.

### Database Analyses

#### Identification of putative Ath_DRM1 and Ath_DRM2 Actinidia homologues

*Arabidopsis thaliana* nucleotide and predicted protein sequences corresponding to Ath_DRM1 (NP_001154378; At1g28330.1) and Ath_DRM2 (NP_850220; At2g33830.1) (http://www.arabidopsis.org) were used to interrogate the Plant & Food Research EST *Actinidia* (sp.) database [23] using BLASTN and TBLASTN, respectively. Reciprocal BLAST Analysis (RBA) was used to identify optimal pair-wise partners.

#### Identification of putative DRM1 homologues in other species

Multiple database searches were performed to identify all putative DRM1 candidates. This was achieved using BLAST programs (TBLASTN and BLASTP) available in association with TAIR, MAtD, TIGR, ExPASy and NCBI plant databases. The nucleotide and/or translated protein sequences, corresponding to *Arabidopsis thaliana* Ath_DRM1 (NP_001154378; At1g28330.1) and Ath_DRM2 (NP_850220; At2g33830.1), were used as the query sequences.

#### Quantitative real time PCR analysis

Gene specific primers were designed using Primer3 [24] (Figure S1) and quantitative real-time PCR (qRT-PCR) reactions were performed as previously described [22]. Data were analyzed on relative quantification monocolour LightCycler® software 4.0 (Roche Diagnostics) and expression normalised to the *Actinidia deliciosa* actin gene (Ade_Actin: FG470439) based upon previously reported low variability of expression and stability index values [22] (Figure S2 A and B).

#### Construction of phylogenetic trees

All multiple alignment analyses were performed with the Geneious Alignment programme as part of the Geneious Pro 5.3.4 software, using an opening penalty of 12 and an extension penalty of 3 (http://www.geneious.com). All sequences used in the DNA alignments represent the putative full length non-redundant contiguous sequences *Actinidia delicios*a: Ade_DRM1_IA; *Actinidia deliciosa^1^*: Ade_DRM1_ID; *Actinidia deliciosa*: Ade_DRM1_IE; *Actinidia deliciosa^1^*: Ade_DRM1_IG; *Actinidia deliciosa^1^*: Ade_DRM1_IIA.1; *Actinidia deliciosa^1^*: Ade_DRM1_IIA.3; *Actinidia deliciosa^1^*: Ade_DRM1_IIA.4; *Actinidia deliciosa*: Ade_DRM1_IID.1; *Actinidia deliciosa*: Ade_DRM1_IID.2; *Actinidia deliciosa:* Ade_DRM1_IID.3 (note: *Actinidia deliciosa* ^1^ represents contiguous sequences composed of both *Actinidia deliciosa* and the closely related *Actinidia chinensis* species). Phylogenetic and molecular evolutionary analyses were performed with Tree Builder programme as part of the Geneious Pro 5.3.4 software using the Jukes-Cantor genetic distance model, inferred by the neighbour-joining method (http://www.geneious.com). The resulting tree topology was evaluated and bootstrap analyses based upon 1000 replicates. Numbers on nodes represent bootstrap values and branches are supported by ≥50% bootstrap values, which are statistically supported. All sequences used in the analyses represent the non-redundant, deduced full length amino acid sequences. The DRM1 conceptual protein translation sequences used in these alignments are listed in Figure S3.

### Bioinformatic analysis of *Actinidia* DRM1 gene family: Primary sequence analyses and disorder prediction

#### Analysis of DRM1 amino acid composition

Intrinsic disorder term is used to describe proteins or segments of proteins that fail to fold into a defined 3D structure and exhibit a particular amino acid composition characterised by the high abundance of disorder-promoting residues. Such proteins, or specific regions (known as intrinsically disordered proteins, IDPs, or intrinsically disordered regions, IDRs), have been shown to be enriched in the eight amino residues: R, D, Q, E, L, M, P and S, referred to as disorder-promoting residues; whilst being depleted in the order-promoting residues of N, C, H, I, L, F, T, W, Y and V. In addition, it is proposed that the amino acid residues A and G should be considered neutral in terms of order/disorder contribution. Amino acid compositional analysis was undertaken by comparing the relative amino acid compositions of i) all putative DRM1s; ii) all putative *Actinidia* DRM1 candidates, iii) all putative Type I plant DRM1 candidates, and iv) all putative Type II plant DRM1 candidates with the relative amino acid compositions of experimentally characterized intrinsically disordered proteins from the Disprot 3.4 database (containing 460 verified IDP entries and 1103 verified disordered regions from the Disprot database [25]. We also directly compared compositions of Type I and Type II plant DRM1 candidates. In this analysis, enrichment or depletion in each amino acid type is expressed as (C_S1_–C_S2_)/C_S2_, i.e., the content of a given residue in a query dataset (C_S1_) relative to the corresponding value in a background dataset (C_S2_). This analysis was done using the Composition Profiler tool [26] available via the internet (http://www.cprofiler.org), utilizing the default parameters to identify amino acids enriched in various disordered proteins (http://www.cprofiler.org/cgi-bin/profiler.cgi) [26].

#### Charge-hydropathy and cumulative distribution function plot

CH-CDF plot is a combined analysis of protein using *C*harge-*H*ydropathy (CH) plot [27] and *C*umulative *D*istribution *F*unction (CDF) plot [27], [28]. CH plot is plotted by the averaged Kay-Doolittle hydrophobicity of a protein (*x*-axis) and averaged net charges of the same protein (*y*-axis). There is a boundary line that separates the CH plot into the up-left region where disordered proteins are normally located and the down-right region where structured proteins are normally located. CDF is a cumulated histogram of disordered residues at various disordered score. The cumulated histogram for structured proteins increases faster in the range of smaller disordered scores and then flattens at larger disordered scores, while the cumulated histogram for disordered proteins increases slightly in the range of lower disordered score but significantly at higher disordered scores. So there is also a boundary line identified in the CDF plot. The distances to the boundary lines in both CH and CDF plots from a specific protein are further used as coordinates on the *x*- and *y*-axes to build up the CH-CDF plot [29]. The disordered scores used for the CDF plot in this study are from PONDR FIT disorder predictor (Predictor Of Natural Disordered Regions) [30].

#### Prediction of order/disorder of Actinidia DRM1 proteins

PONDR FIT [30] was used to predict the order/disorder of DRM1 proteins bioinformatically. PONDR FIT outputs are represented by real numbers between 1 and 0, where 1 is the ideal prediction of disorder and 0 is the ideal prediction of order. A default threshold was applied with disorder assigned to values greater than or equal to 0.5.

#### Prediction of secondary structure and solvent accessibility of DRM1 proteins

Secondary structure prediction (α-helix; β-sheet and random coil) and solvent accessibility were undertaken using the web-based predictive program Jpred3 incorporating Jnet (Jnet version 2.2 UniRef90 Release 15.4)

(http://www.compbio.dundee.ac.uk/www-jpred) [31], [32].

#### Identification of putative α-MoRFs in DRM1 predicted protein sequences

Molecular recognition features (MoRFs) are short binding regions located within longer intrinsically disordered regions that bind to protein partners via disorder-to-order transitions. In the case of α-MoRFs, such binding results in the generation of an α-helix conformational change in the MoRF itself. Identification of potential α-MoRFs was determined using a α-helix-forming molecular recognition feature predictor (α-MoRFs-II) based on PONDR FIT prediction and a large positive data set [33], [34].

#### Prediction of disulphide bonding; non-regular secondary structure (NORSp); nuclear localisation sequence (NLS); protein globularity; protein-protein binding and protein-DNA binding

The prediction of potential disulphide bonding NOn-Regular Secondary structure (NORSp), nuclear localisation sequence (NLS); Protein-Protein binding and Protein-DNA binding was undertaken using the PredictProtein server (Technischen Universität Mϋnchen (TUM), http://www.predictprotein.org) with default parameters.

#### Prediction of putative PEST proteolytic cleavage sites

Putative proteolytic cleavage sites identified by the PEST motif were identified using epestfind (http://mobyle.pasteur.fr/cgi-bin/portal.py?#forms::epestfind), which allows for the rapid and objective identification of PEST motifs in protein target sequences. PEST motifs are defined as hydrophilic stretches of at least 12 amino acids in length with a high local concentration of critical amino acids.

#### Prediction of potential phosphorylation sites of DRM1

Predicted phosphorylation potential was undertaken using the web-based predictive program DEPP (Disorder Enhanced Phosphorylation Predictor) as part of the PONDR website (http://www.pondr.com), with default parameters applied. Access to PONDR FIT was facilitated by Molecular Kinetics (Indianapolis, IN).

## Results

### Bioinformatic analyses

#### DRM1 family proteins are highly conserved within kiwifruit and within other plant species

Several independent, putative full length *Actinidia* DRM1 candidates have been identified using reciprocal blast analyses (RBA) with the predicted protein sequence Ath_DRM1 as the query sequence. The *Actinidia* putative full length cDNA clones consist of 574–653 bp, contain predicted ORFs in the region of 348–363 bp, with all in-frame termination codons annotated as a TGA stop codon. The 5′ UTR sequence is relatively short in all candidates (≤ 41 bp); with the 3′ UTR sequence ranging from 200 to 276 bp. The conceptual translations of the *Actinidia* gene models yield predicted proteins between 116 and 121 aa with calculated molecular weights of approximately 12.6–13.3 KDa (Figure S4). No organellar targeting, NLS or signal peptide sequences were identified, as previously observed for Eum_ARP1 [19].

**Figure 1:**
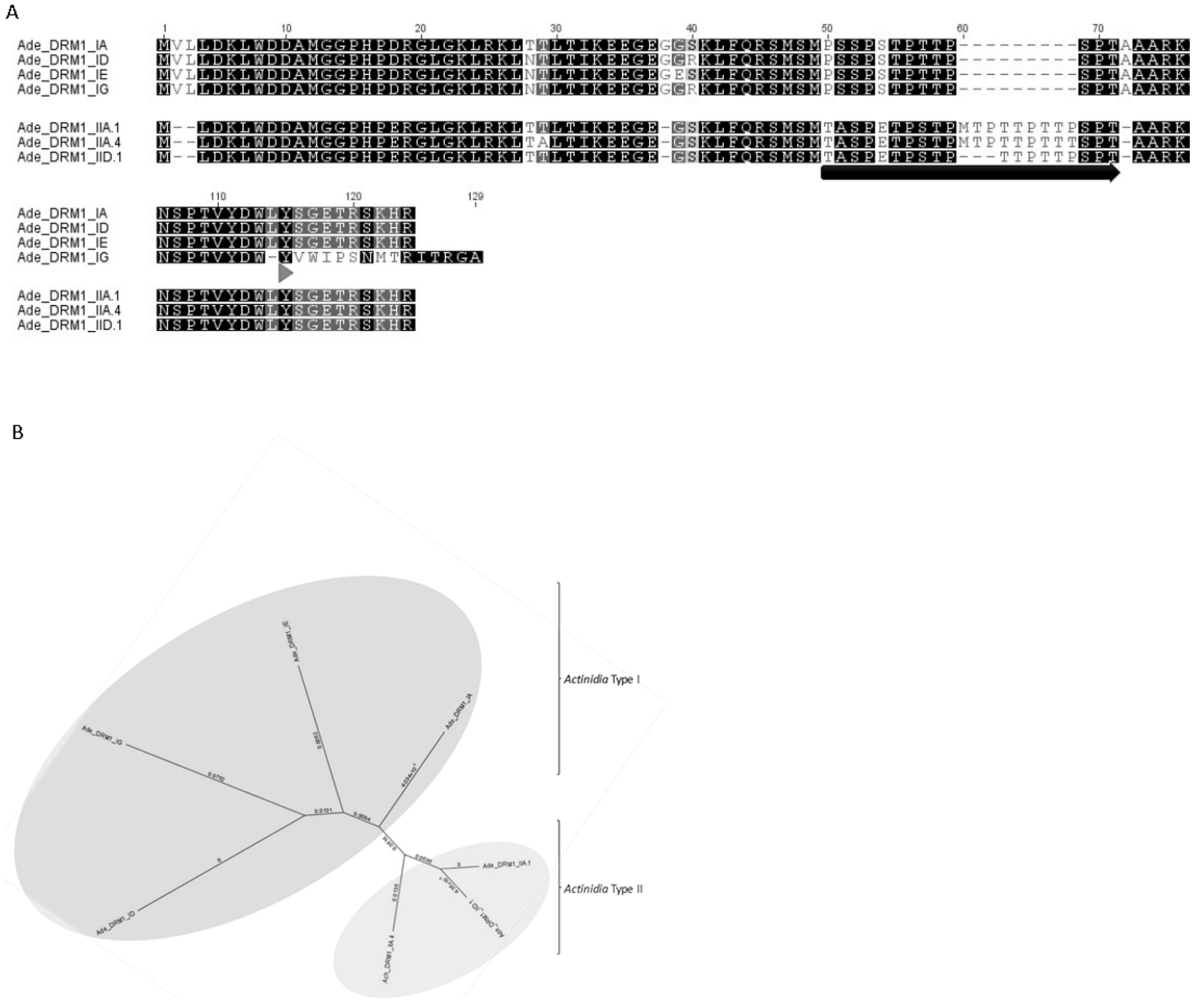
Multiple sequence alignment and phylogenetic tree analysis of *Actinidia* DRM1 family proteins. (A): Multiple sequence alignment and (B) phylogenetic tree analysis of *Actinidia* DRM1 family proteins *Actinidia deliciosa*: Ade_DRM1_IA; *Actinidia deliciosa^1^*: Ade_DRM1_ID; *Actinidia deliciosa*: Ade_DRM1_IE; *Actinidia deliciosa^1^*: Ade_DRM1_IG; *Actinidia deliciosa^1^*: Ade_DRM1_IIA.1; *Actinidia deliciosa^1^*: Ade_DRM1_IIA.4; *Actinidia deliciosa*: Ade_DRM1_IID.1. →P/T rich region; ▸Alternative splice site. *Actinidia deliciosa^1^*: non-redundant contiguous sequences contain both *Actinidia deliciosa* and *Actinidia chinensis* expressed sequence tag (EST) sequences.

Multiple sequence alignment combined with the phylogenetic analysis (Geneious Pro 5.3.4) using non-redundant predicted protein sequences highlight the presence of two distinct *Actinidia* DRM1 families: Type I and Type II, represented by four and three independent gene models, respectively (Figure 1B). Single-nucleotide polymorphisms (SNPs) within the Type I and Type II families account for the intra- and inter-family variation with resultant mis-sense mutations observed. Such mutations predominately occur in the non conserved, variable mid-region. *Actinidia* Type I candidates share 93.5% pairwise identity at the amino acid level within their own family, reaching a maximum of 98.3% when analysed in the absence of Ade_DRM1_Type IG. *Actinidia* Type II candidates also share high sequence identity within the Type II family, 98% at the amino acid level. Sequence identity across the two *Actinidia* families is less, but still displays a high level of conservation, with 84.9% pairwise identity observed. *Actinidia* Type II DRM1 candidates are distinct from Type I based upon a deletion of the two hydrophobic amino acids V and L at the N-terminal and an insertion of a small 6–8 aa P and T rich region. Of note is the Ade_DRM1_Type IG, which has a deduced protein sequence of 119 aa cf. 115 aa of all other *Actinidia* DRM1 Type I candidates. Alignment of Ade_DRM1_IG cDNA sequence with the *Actinidia chinensis* DRM1 gDNA sequence indicates that this is the consequence of an alternative splicing event resulting in the retention of the second intron present in the coding region of the DRM1 gene, with the exploitation of a downstream in-frame alternative stop codon (TGA) compared with the other Type I members; resulting in a mature transcript with an extended 3′ UTR (Figure 1A and Figures S4 and S5). Alignment of the *Actinidia chinensis* DRM1 genomic sequence and Ade_DRM1_1G (Figure S5) reveals the presence of two introns within the open reading frame (ORF) of the DRM1 gene. Analysis of the *Actinidia* cDNAs and predicted protein sequences suggests that Ade_DRM1_IG is most closely related to the fully processed Ade_DRM1_ID. Ade_DRM1_IG, the alternatively spliced transcript, is associated with tissues exposed to HC or abiotic stress known to initiate a sub-lethal stress response.

**Figure 2:**
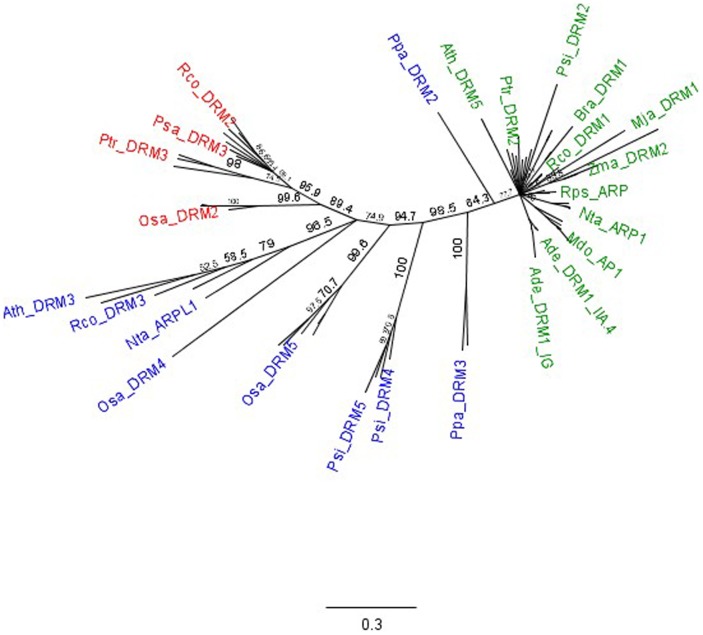
Phylogenetic tree analysis of plant-specific DRM1 family proteins. Representative clade members are annotated as follows: Clade 1: green; Clade 2a: red and Clade 2b: blue. *See Figure S3 for all predicted protein sequences and Figure S6 for multiple sequence alignment of all candidates used in phylogenetic analysis.*

Multiple protein sequence alignment using conceptual translations of the kiwifruit and additional taxonomically diverse plant DRM1 candidates highlight the presence of two distinct domains associated with this gene, as previously reported with smaller datasets [18], [19]. Clade 1 is represented by members with both a highly conserved N-terminal domain (Domain I) and highly conserved second domain (Domain II). Clade 2 members, on the other hand, exhibit only the single highly conserved Domain I. However, based upon the additional DRM1 candidates identified through the gene mining approach used in this study, it is apparent that there has been an extension of Clade 2, encompassing additional DRM1 candidates that possess a partially conserved Domain II (Figure 2, Figure S6). *Actinidia* DRM1 putative homologues display high protein sequence identity with Clade 1 members such as Psa_DRM1 (64.7%). Sequence identity is also observed with Clade 2a members, but to a lesser degree (cf. Sbi_DRM2: 42.1%) and less sequence identity with Clade 2b members (cf. Sbi_DRM1: 40.3%).

A putative *Actinidia* homologue for Ath_DRM2, a closely related Ath_DRM1 family member, has not been identified, despite reiterative blast analyses. This was also observed in apple (*Malus* x *domestica* ‘Golden Delicious’) and avocado (*Persea americana)*.

### Predicted properties and secondary structure of *Actinidia* DRM1 protein

**Figure 3:**
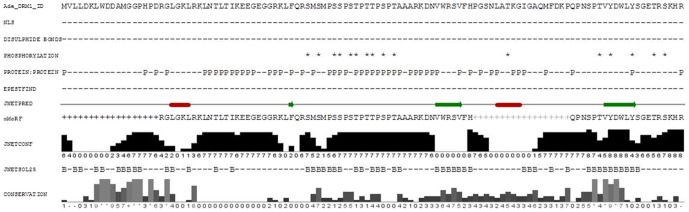
Predicted Properties and Secondary Structure of *Actinidia* DRM1 proteins. Composite diagram illustrating predicted secondary structures and properties of *Actinidia* DRM1 proteins, using Ade_DRM1_ID as a representative example. Potential nuclear localisation signal (NLS), disulphide bonds, phosphorylation and protein:protein interaction sites generated via PredictProtein (http://www.predictprotein.org). The secondary structure prediction, JNETPRED, generated by Jpred3/JNet (http://www.compbio.dundee.ac.uk/www-jpred) [31], [32] is shown below. The black cylinder represents predicted α-helices and black block arrows represent β-strands. The bar chart and associated numbers in JNETCONF illustrate the prediction confidence on a scale of 0–9. The CONSERVATION data suggest positions within the alignment where the physico-chemical properties of the amino acids are most highly conserved. Solvent accessibility (JNETSOL25) is annotated with a B for solvent accessibility (buried) or a short line for solvent accessibility at a 25% cut-off. Predicted putative PEST proteolytic cleavage sites were determined using EPESTFIND (epestfind (http://mobyle.pasteur.fr/cgi-bin/portal.py?#forms::epestfind) (? presence; – absence of predicted PEST site). Putative α-MoRF motifs were determined using α-MoRFs-II based on PONDR FIT prediction and a large positive data set [33], [34] (+ α-MoRFs motif I and + α-MoRFs motif II).

The predicted secondary structures of the *Actinidia* DRM1 family was determined. For simplicity, Ade_DRM1_1D is shown as a representative of the entire family. Overall, the *Actinidia* DRM1 predicted protein family appear to be non-globular in structure, with no α-helices or coiled coils confidently predicted and with only one β-strand prediction, supported with JNETCONF data (residues 101 – 106, inclusive) associated with the conserved C-terminal domain (Figure 3). In total, two conserved domains are evident: namely an N-terminal domain (Domain I; residues 1 – 26) and a second domain (Domain II; residues 45 – 116) (Figure 3 and Figure S7). The *Actinidia* DRM1 proteins possess no C residues, which have been shown to play a major role in the folding process of many proteins via the formation of stabilising disulphide bridges and indeed, this lack of C residues may also suggest an intracellular localisation of the mature protein. In addition, no transmembrane or nuclear localisation signal (NLS) protein signatures were detected. Several predicted protein:protein interaction sites were identified generally associated with the mid-region of the *Actinidia* DRM1 proteins, aligning with a predicted phosphorylation region (Figure 3). The predicted phosphorylation sites highlighted (DEPP, http://www.pondr.com) (Figure 3), correlate with the S, P and T-rich regions observed in both *Actinidia* Type I and Type II family members (Figure 1). In particular, conservation of residues T^55^ and T^57^ is observed compared with the Ptr_DRM2 homologue at residues T^61^ and T^63^, recently shown to be phosphorylated in dormant buds of poplar [35]. The presence of methionine residues in the immediate vicinity of the predicted phosphorylation sites M^47^ and M^49^ (Figure 3) suggests a potential additional degree of protein regulation via methionine oxidisation, which has recently been implicated in a stress-induced ROS-mediated signalling cascade in plants [36]. In addition, the presence of proline residues also in the immediate vicinity of the predicted phosphorylation sites P^50^, P^53^, P^56^, P^59^ and P^61^ (Figure 3) suggests a putative involvement in transient protein:protein interactions, known to be regulated by phosphorylation, and has been proposed to have a role in the prevention of protein aggregation [37].

### Actinidia DRM1 family is predicted to be intrinsically disordered

#### Amino acid composition of the Actinidia DRM1 family

**Figure 4:**
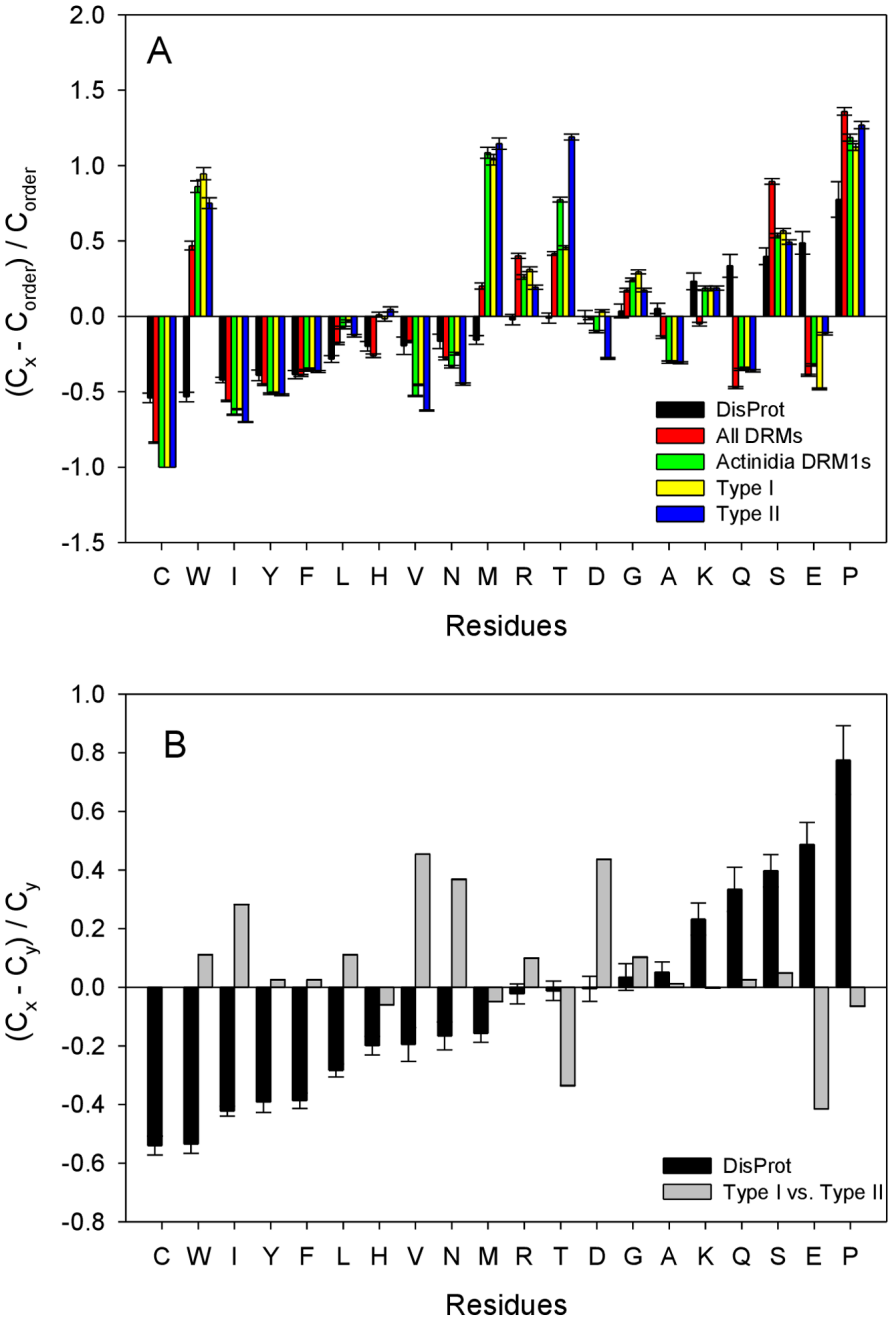
Compositional profiling of DRM1 proteins. A. Fractional difference in amino acid composition between the various sets of DRM1 proteins: all the DRM1 candidates from available plant species (red bars), the combined kiwifruit DRM1 protein family (green bars), Type I DRM1 proteins (yellow bars), and Type II DRM1 proteins (blue bars). Composition profiles of typical intrinsically disordered proteins from the DisProt database are shown for comparison (black bars). The fractional difference in composition between a given protein set and a set of completely ordered proteins was calculated for each amino acid residue as (C_x_-C_order_)/C_order_, where C_x_ is the content of a given amino acid in a given protein set, and C_order_ is the corresponding content in the fully ordered dataset. B. Fractional difference in amino acid composition between the members of the Type I and Type II DRM1 proteins (grey bars). Composition profiles of typical intrinsically disordered proteins from the DisProt database are shown for comparison (black bars). The fractional difference was evaluated as (C_x_-C_y_)/C_y_, where C_x_ is the content of a given amino acid in a query set of proteins (set of Type I DRM1 proteins, or set of disordered proteins from DisProt database), and C_y_ is the corresponding content in a background set of proteins (set of Type II DRM1 proteins, or set of ordered proteins).

Ade_DRM1 proteins are predicted to be small (<15 KDa) and highly conserved, with no known structural or functional domains, as indicated above. This lack of known structural and functional domains suggests a potential intrinsically disordered nature of the Ade_DRM1 proteins. Recently, there has been growing interest in such protein intrinsic disorder which is becoming increasingly recognized in proteomics research [21]. The amino acid compositions of all seven full-length *Actinidia* DRM1 candidates were analysed to determine if DRM1 predicted protein sequences adhere to this general observation. To validate this observation, an initial comparison of the amino acid composition of typical IDPs from the Disprot 3.4 database (containing 460 verified IDP entries and 1103 verified disordered regions) [25] with the composition of ordered proteins from the PDB database was undertaken (see black bars Figure 4, plots A and B). Here, the fractional difference in composition between a given protein set and a set of completely ordered proteins was calculated for each amino acid residue. The fractional difference was evaluated as (C_x_-C_order_)/C_order_, where C_x_ is the content of a given amino acid in a given protein set, and C_order_ is the corresponding content in the fully ordered dataset [26], [38]. In this presentation, negative values correspond to residues that are depleted in a given dataset in comparison with a set of ordered proteins, whereas the positive values correspond to the residues which are over-represented in the set. This analysis revealed expected biases in variation of average amino acid residue frequencies of disordered proteins in comparison with the average frequencies found in the ordered globular proteins from the Protein Data Bank.

Figure 4A illustrates that in a manner similar to the IDPs from the DisProt 3.4 data set [25] the combined *Actinidia* DRM1 Type I and Type II protein family showed an enrichment in the major disorder-promoting residues (R, T, K, S and P) and an associated depletion in the major order-promoting amino acids (e.g. C, I, Y, F and V). Comparable behaviour is obvious for the members of the Type I and Type II families of *Actinidia* DRM1 proteins. Overall, the compositional profiles of *Actinidia* DRM1 proteins (both as an entire family and as members of individual types) are very typical for IDPs, with typical deviations from the behaviour of the averaged IDPs being an enrichment in order-promoting W and depletion in disorder-promoting Q and E residues.

Analogous composition profile-based analysis of the Type I and Type II *Actinidia* DRM1 proteins (where the fractional difference was evaluated as (C_x_-C_y_)/C_y_, where C_x_ is the content of a given amino acid in a set of Type I *Actinidia* DRM1 proteins, and C_y_ is the content of the same amino acid in a set of Type II *Actinidia* DRM1 proteins), revealed that in comparison with Type II proteins, Type I *Actinidia* DRM1 proteins contain more I, N, M, and D, and are depleted in T and E (see Figure 4B). However, none of these differences was statistically significant according to the two-sample t-test incorporated into the Composition Profiler webpage (http://www.cprofiler.org/cgi-bin/profiler.cgi). Here, a particular enrichment or depletion is statistically significant when p-value (the lowest value at which the null hypothesis that the same underlying Gaussian distribution generated both samples can be rejected), is lower than or equal to a statistical significance (alpha) value (0.05), which was not the case for any residue of the analyzed Type I and Type II *Actinidia* DRM1 proteins.

#### Prediction of protein structural disorder

**Figure 5:**
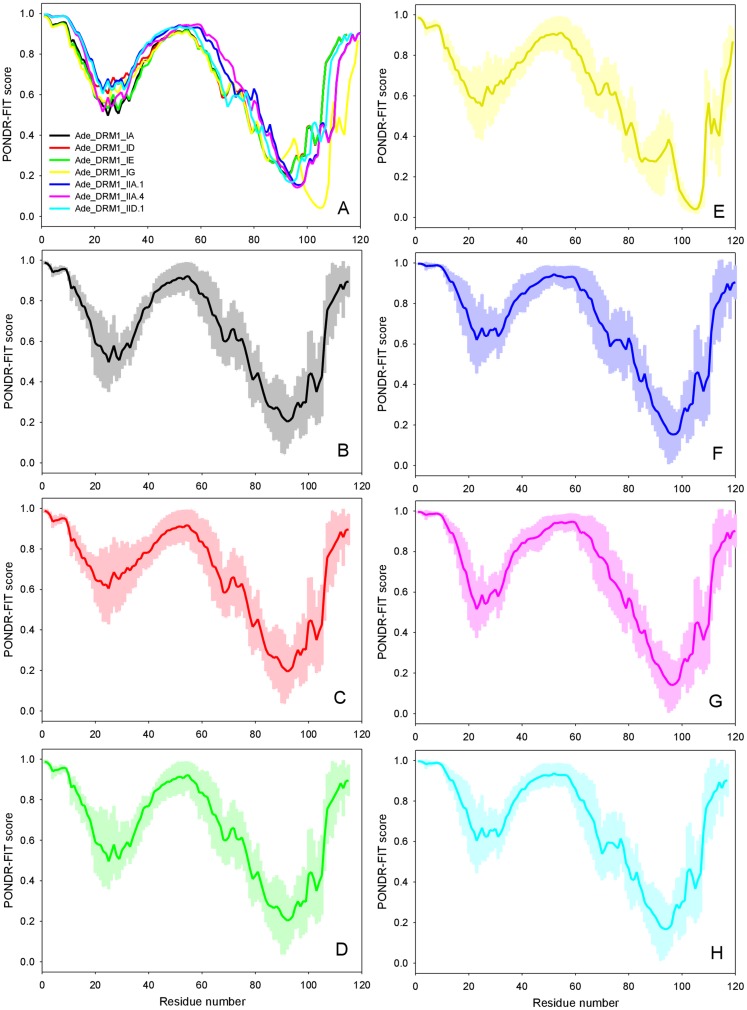
Analysis of disorder distribution profiles in members of the *Actinidia* DRM1 family proteins. A. Combined representation of disorder profiles for all the members of *Actinidia* DRM1 protein family; B – H. Disorder profiles for individual members. Shaded areas around the curves correspond to the distribution of the errors in the determination of the intrinsic disorder propensities by PONDR FIT algorithm.

Prediction of protein structural disorder based upon primary amino acid sequence was undertaken using PONDR FIT intrinsic disorder predictor [30]. In this study, PONDR FIT was chosen since this meta-predictor is among the more accurate disorder predictors [30]. The PONDR FIT score patterns of all the *Actinidia* DRM1 proteins are similar to one another and are characterized by the high intrinsic disorder content (Figure 5A). Disorder distribution profiles for the individual members of the *Actinidia* DRM1 family members are shown in Figure 5B-H. Shaded areas around the curves correspond to the distribution of the errors in the determination of the intrinsic disorder propensities. An independent orthogonal predictor of potential protein disorder, namely METADISORDER (predictprotein.org), and NORS2st were used to validate the PONDR FIT data. The entire *Actinidia* DRM1 family was predicted to be disordered to some degree.

**Figure 6:**
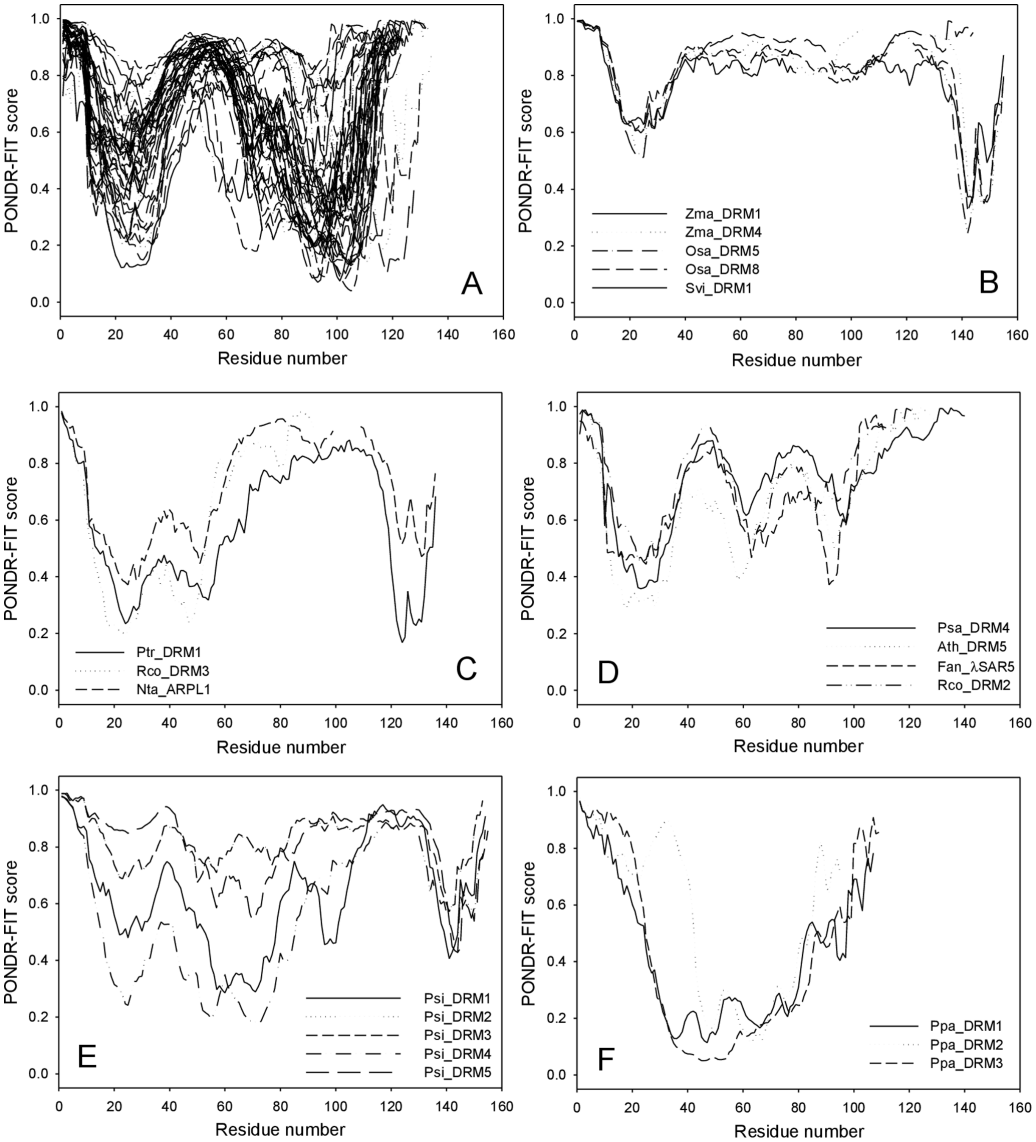
Analysis of disorder distribution profiles associated with plant DRM1 family proteins. Plant DRM1 family proteins are organised into six distinct classes based upon the similarity of their disorder profiles A – F.

Figure 6 represents the result of the similar analysis for all the plant DRM1 family proteins used to build the sequence alignments shown in Figure S6 and clearly shows that all these proteins are predicted to be highly disordered. The proteins can be grouped into six classes according to the similarity of their disorder profiles, with the largest group possessing the disorder profiles similar to those of the *Actinidia* DRM1 proteins (i.e., characterized by two minima in the vicinity of residues 20 and 100, see Figure 6A). Zma_DRM1, Zm1_DRM4, Osa_DRM5, Osa_DRM6, and Sbi_DRM1 are characterized by two minima, located in the close proximities to their N- and C-termini (Figure 6B), whereas Ptr_DRM1, Rco_DRM3, and Nta_ARPL1 have a double minimum in the N-terminal region (around residues 20 and 50), with Ptr_DRM1 and Nta_ARPL1 possessing another minimum in their C-termini (Figure 6C). There are three well-defined minima centred at residues 20, 60 and 100 for Psa_DRM4, Ath_DRM5, Fan_λSR5 and Rco_DRM2 (Figure 6D). Members of the Psi_DRM family are characterized by the pronounced minimum at their far-most C-terminal parts (around residue 140) and differently developed minima in the N-terminal and central parts (Figure 6E). Finally, Psa_DRM proteins possess a broad, but shallow minimum in their central regions (Figure 6E). Irrespective of the described peculiarities in the per-residue disorder distributions, all plant DRM1 proteins are excessively disordered.

**Figure 7:**
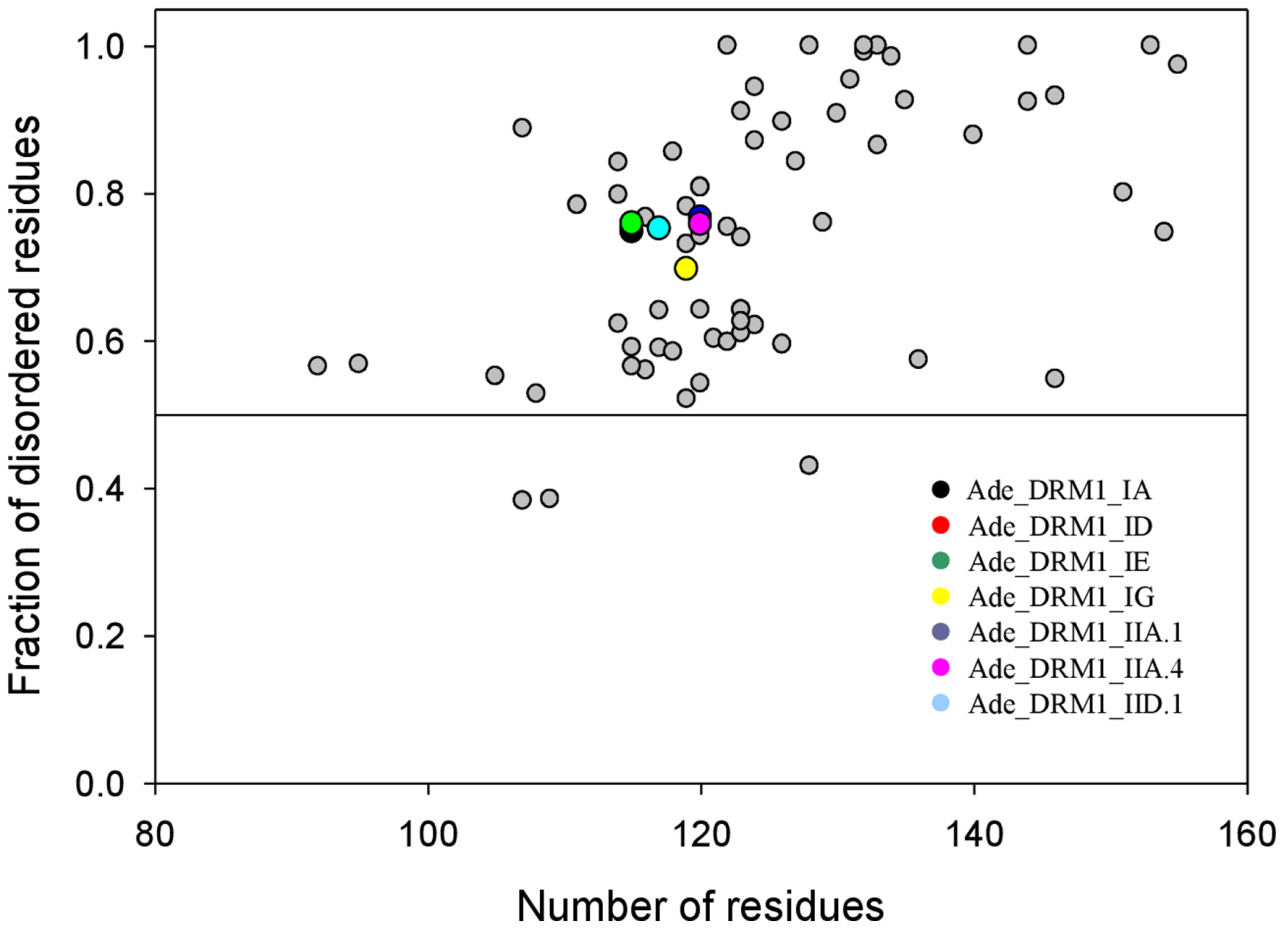
Determination of the overall disorder predisposition in *Actinidia* DRM1 family proteins. Data are presented as the dependence of the fraction of disordered residues upon protein length.

This conclusion is further supported by the evaluation of the overall disorder predisposition of these proteins based on the estimation of the fractions of their residues predicted to be disordered. Figure 7 represents the result of this analysis in the form of the dependence of the fraction of disordered residues on protein length and clearly shows that 69 of 72 proteins are predicted to be mostly disordered, since the majority of their resides possess disorder scores above the 0.5 threshold. Figure 7 also shows that approximately 80% of residues in all 7 *Actinidia* DRM1 proteins are disordered.

#### CH-CDF analysis

**Figure 8:**
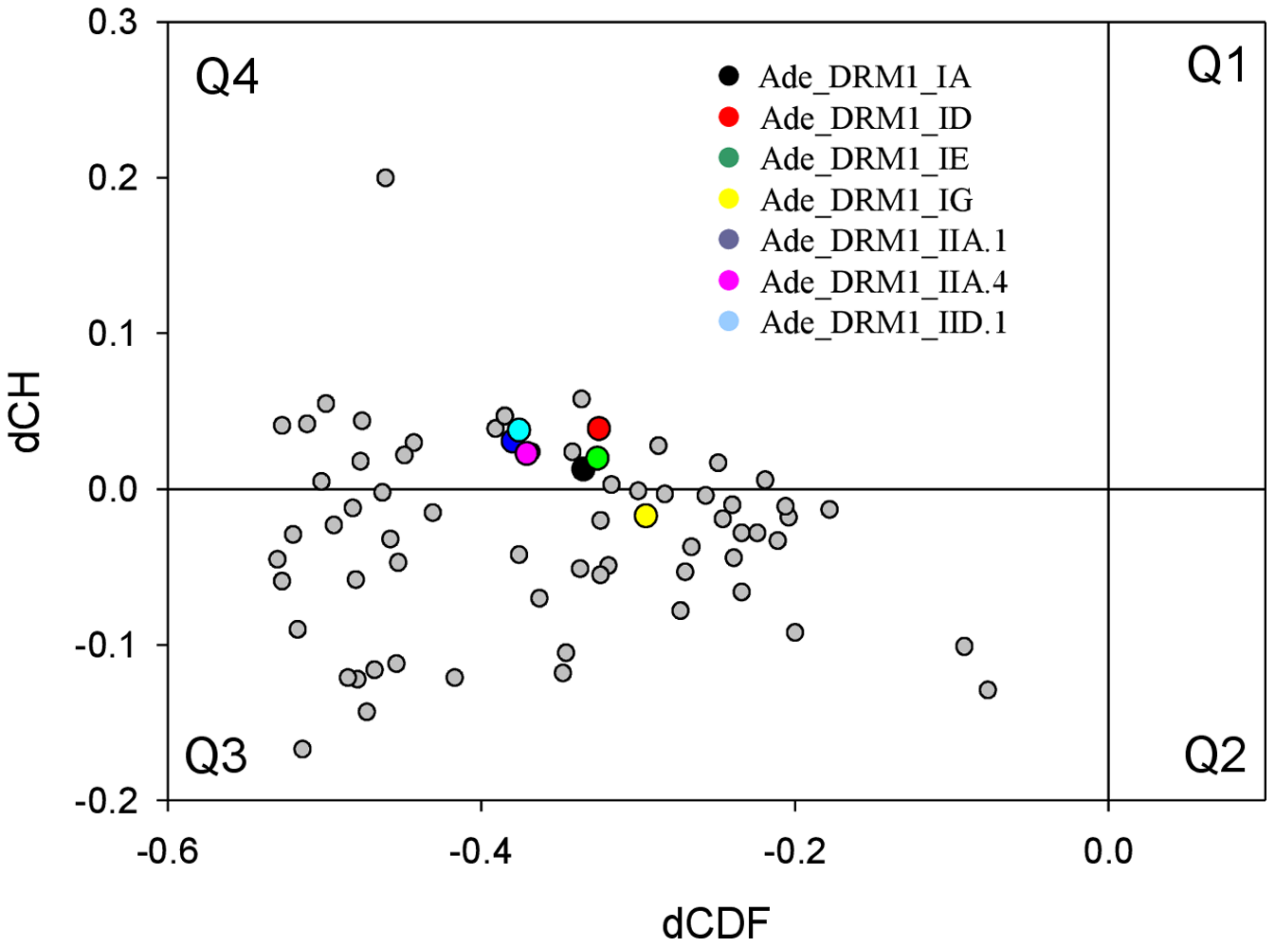
Disorder analysis: combined CH-CDF plot of plant DRM1 proteins. The CH-CDF plot is sectioned into four distinct quadrants as previously described [29]. Quadrant 1 (Q1) rare proteins: predicted to be disordered by CH and ordered by CDF analyses; quadrant 2 (Q2) ordered proteins: predicted to be ordered proteins by CH and CDF analyses; quadrant 3 (Q3) mixed proteins: predicted to be ordered proteins by CH and disordered proteins by CDF comprising of proteins that contain both ordered and disordered regions; and quadrant 4 (Q4) disordered proteins: predicted to be disordered by both CH and CDF analyses. *y* axis: distance of each DRM1 protein from major boundary line in CH plot (positive for disordered, negative for ordered or small number of disordered); *x* axis: distance of each DRM1 protein from boundary line in CDF plot (positive for ordered, negative for disordered).

Figure 8 provides further support to the mostly disordered nature of the plant DRM1 proteins, representing the results of their CH-CDF analysis. In this plot, the coordinates of each spot are calculated as a distance of the corresponding protein in the CH-plot (charge-hydropathy plot) from the boundary (Y-coordinate) and an average distance of the respective cumulative distribution function (CDF) curve from the CDF boundary (X-coordinate) [29]. The primary difference between these two binary predictors (i.e., predictors which evaluate the predisposition of a given protein to be ordered or disordered as a whole) is that the CH-plot is a linear classifier that takes into account only two parameters of the particular sequence (charge and hydropathy), whereas CDF analysis is dependent on the output of the PONDR FIT predictor, a nonlinear classifier, which was trained to distinguish order and disorder based on a large feature space. According to these methodological differences, CH-plot analysis is predisposed to discriminate proteins with a substantial amount of extended disorder (random coils and pre-’molten globules’) from proteins with compact conformations (‘molten globule’-like and rigid well-structured proteins). On the other hand, PONDR FIT-based CDF analysis may discriminate all disordered conformations, including molten globules, from rigid well-folded proteins [27]. Therefore, this discrepancy in the disorder prediction by CDF and CH-plot provides a computational tool to discriminate proteins with extended disorder from ‘molten globules’. Positive and negative Y values in Figure 8 correspond to proteins predicted within CH-plot analysis to be extended or compact, respectively. In contrast, positive and negative X values are attributed to proteins predicted within the CDF analysis to be ordered or intrinsically disordered, respectively. Thus, the resultant quadrants of CH-CFD phase space correspond to the following expectations: Q1, proteins predicted to be disordered by CH-plots, but ordered by CDFs; Q2, ordered proteins; Q3, proteins predicted to be disordered by CDFs, but compact by CH-plots (i.e., putative ‘molten globules’); Q4, proteins predicted to be disordered by both methods. Figure 8 shows that all the members of the plant DRM1 family of proteins can be grouped into two classes related to their localization within the CH-CDF phase space. Here, 25 DRM1 proteins including *Actinidia* Ade_DRM1_IA, Ade_DRM1_ID, Ade_DRM1_IE, Ade_DRM1_IIA.1, Ade_DRM1_IIA.4, and Ade_DRM1_IID.1 proteins are expected to behave as native coils or native pre-molten globules (i.e., to possess low levels of regular secondary structure and be substantially non-compact). All other members of this family including the *Actinidia* DRM1_1G protein are predicted to behave as potential native molten globules (i.e., to possess well-developed secondary structure, hydrophobic core, high compactness degree, and lack of rigid 3D structure).

#### α-helix-forming MOlecular Recognition Features (α-MoRFs) prediction

Often, disordered regions contain local regions with a strong tendency to become ordered. These regions might undergo coupled folding and binding resulting from their interaction with corresponding binding partners. Furthermore, predictions of local order within long disordered regions (which are seen as short downward spikes of predicted order within regions of disorder) often coincide with potential binding sites. These observations constitute a foundation of an algorithm that identifies molecular recognition features (MoRFs) as short regions with increased order propensity and high α-helix-forming propensity, which are located within the long disordered regions and undergo coupled binding and folding of short regions [33], [34]. A systematic application of this predictor to large protein databases indicated that α-MoRFs are very common in proteins with long disordered regions and are likely to play important roles in protein:protein interactions involved in signalling events [33]. Figure S7 shows that most plant DRM1 proteins contain at least one α-MoRF. Among the *Actinidia* proteins, Ade_DRM1_IG has one α-MoRF, Ade_DRM1_ID and Ade_DRM1_IE have two α-MoRFs each, and there are three putative α-MoRFs in Ade_DRM1_IA, Ade_DRM1_IIA.1, Ade_DRM1_IIA.4, and Ade_DRM1_IID.1. The high abundance of α-MoRFs within the members of the plant DRM1 family suggests that these disorder-based features may be utilized by DRM1 proteins for their interactions with binding partners.

### Ade_DRM1_IG mRNA expression is strongly correlated with endodormancy in kiwifruit buds over winter

**Figure 9:**
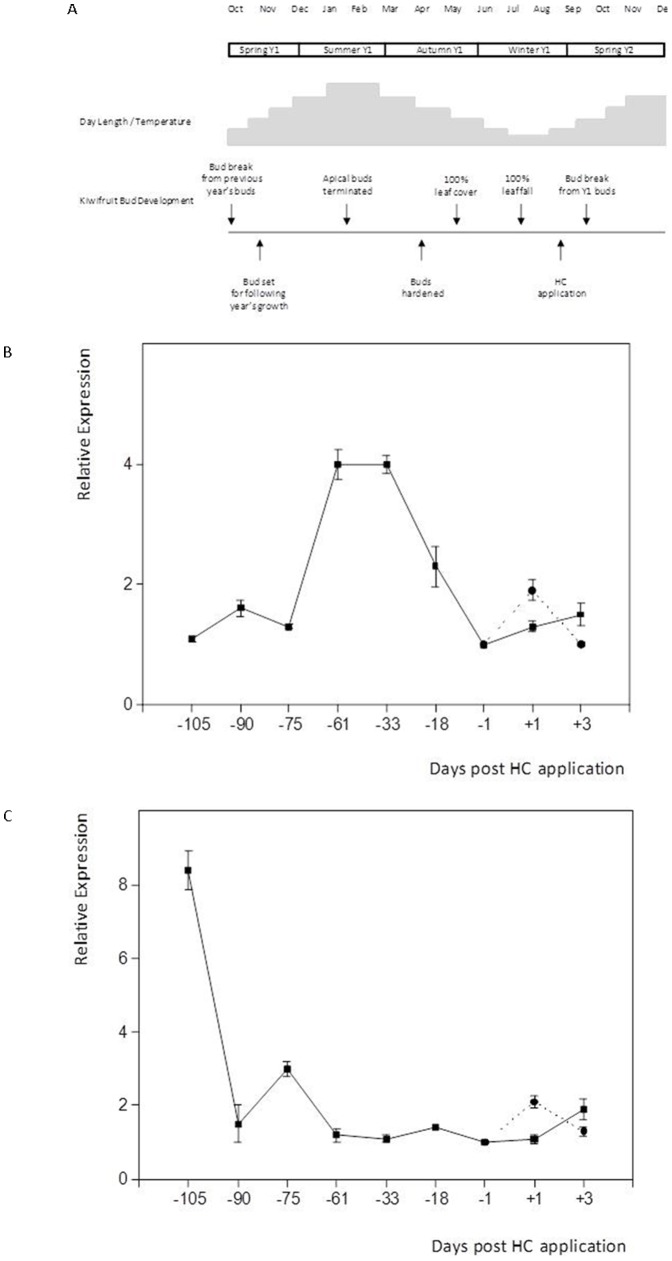
Ade_DRM1_IG mRNA expression profile strongly correlates with endodormancy in whole upward-facing kiwifruit buds over winter. Schematic diagram of mature kiwifruit plant (*Actinidia deliciosa*) growth events (A); mRNA expression profile of Ade_DRM1_IG (B) and Ade_CDKB (C) during endodormancy. ····•···· HC applied —▪— No HC applied.

Southern hemisphere winter and the associated dormancy of kiwifruit buds occur between June and August in any given year (Figure 9A). The Ade_DRM1_IG mRNA expression profile was determined in whole over-wintering kiwifruit buds (May 2005–August 2005) and showed that Ade_DRM1_IG transcript levels began to rise as the leaves abscised, reaching a 4-fold increase by Day -61 (23 June 2005) and were maintained until Day -33 (21 July 2005), by which time 100% leaf drop had been achieved. After that time, Ade_DRM1_IG transcript levels began to drop and reached pre-winter levels at Day -1 (22 August 2005). During the same period of time, the transcript levels of Ade_CDKB mRNA expression showed their greatest decline between the first two time points (−105 and −90) (Figure 9C) suggesting the transition of the buds into the endodormant state, characterised by low mitotic and cell growth activity, had been initiated.

### Addition of HC results in the accelerated loss of Ade_DRM1_IG mRNA expression

**Figure 10:**
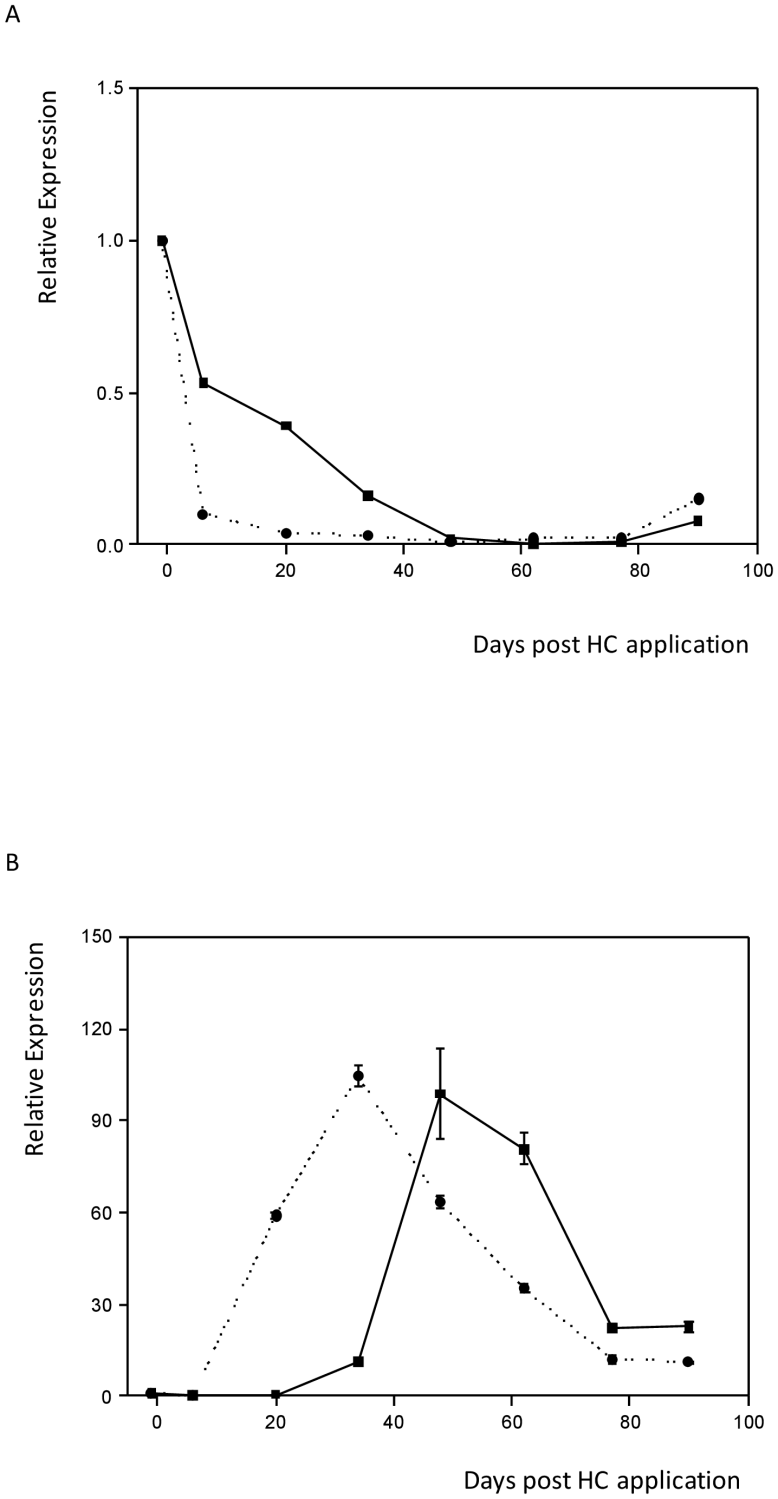
Ade_DRM1_IG expression is rapidly down-regulated upon application of HC in whole kiwifruit buds. Effect of treatment with hydrogen cyanamide (HC) following release of ecodormancy in whole kiwifruit buds upon mRNA expression of (A) Ade_DRM1_IG and (B) Ade_CDKB. ····•···· HC applied —▪— No HC applied.

The addition of HC to accelerate bud outgrowth in kiwifruit is well documented and is a routine tool used by the kiwifruit industry in New Zealand. The effect of HC upon Ade_DRM1_IG mRNA expression profile within whole kiwifruit buds was investigated. Gene-specific primers indicated that the rate of Ade_DRM1_IG expression decreased more rapidly following the application of HC compared to the NHC control (see Figure 10A Day +6 post HC application). As Ade_DRM1_IG transcript levels decreased, there was a concomitant increase in Ade_CDKB mRNA expression, which indicates a reactivation of mitosis and cell growth (Figure 10B).

### *Actinidia* distal buds exhibit pronounced repression of Ade_DRM1_IG mRNA expression compared with basal buds

**Figure 11:**
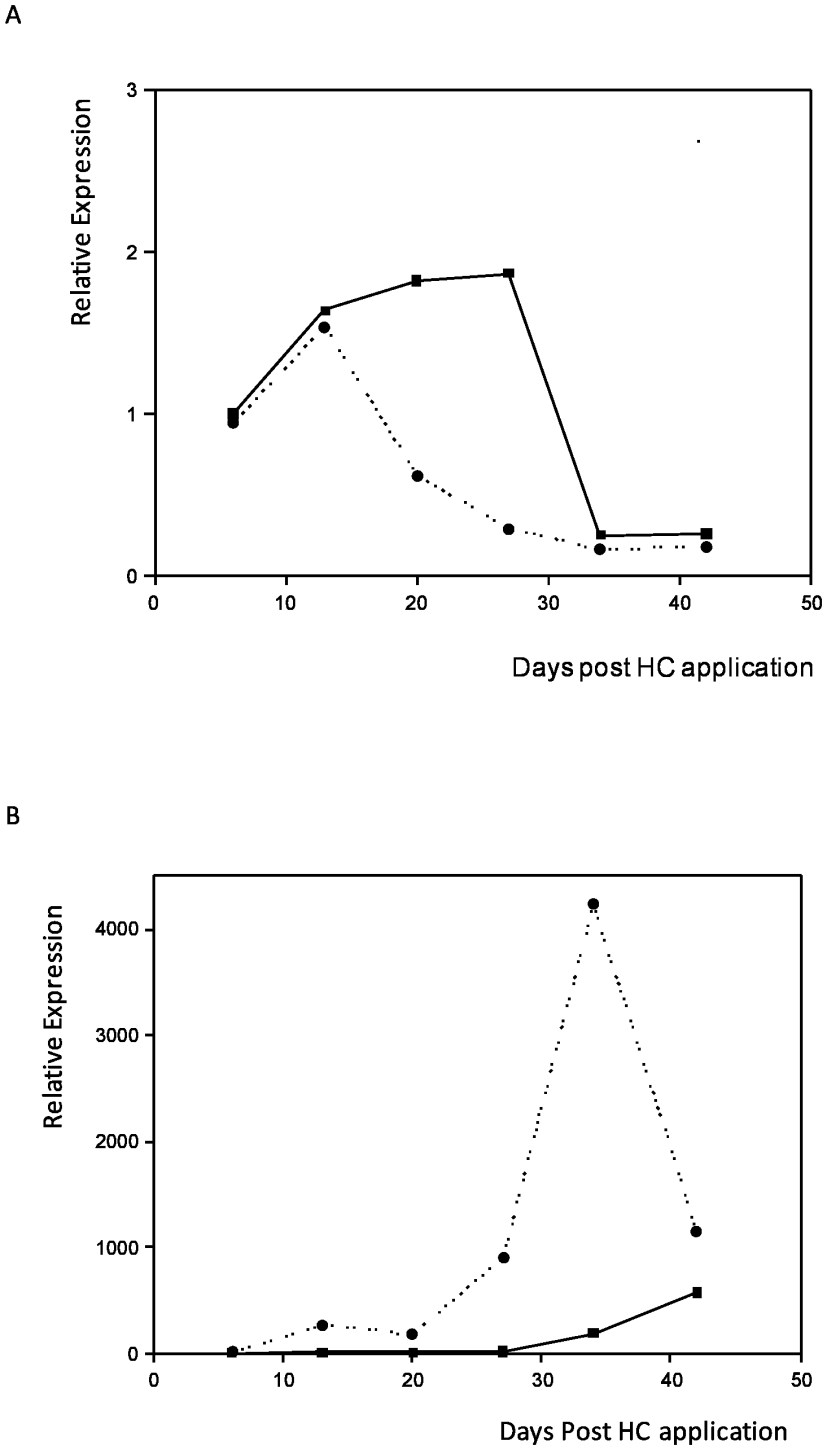
Differential expression of Ade_DRM1_IG is observed in a kiwifruit cane. Effect of bud location within a cane upon Ade_DRM1_IG DRM1 expression.Distal bud: terminal upward-facing bud on cane; Basal bud: upward-facing bud four buds down from the distal bud described above.(A) Ade_DRM1_IG and (B) Ade_CDKB. *(N.B. No HC treatment and all buds upwards facing only). *····•···· Distal bud —▪— Basal bud.

The progression of budbreak in kiwifruit canes following winter dormancy has been well documented to occur in a distal - basal manner, with buds more distal to the main trunk tending to break sooner, particularly during relatively warm winters. An analysis of the DRM1 mRNA expression profile indicated a strong correlation with the loss of Ade_DRM1_IG mRNA expression before budbreak (Figure 11A). Concomitant with this decrease in DRM1 mRNA expression, Ade_CDKB transcript expression increased more rapidly relative to the basal buds, supporting the earlier growth observed in the distal buds. Ade_DRM1 mRNA levels decreased in both distal and basal buds with the later days +34 and +42, correlating to observable budbreak in distal buds only.

## Discussion

The DRM1 gene family is small, but highly conserved and unique to plants. The genes code for a relatively small protein product <20 Kda and have previously been shown to possess two conserved domains. In this paper, it is reported that the DRM1 gene family in *Actinidia* represents a previously unreported family of plant-specific intrinsically disordered proteins. Bioinformatic analysis of Plant & Food Research's *Actinidia* EST database has identified several putative DRM1 gene candidates. Further subdivision of the *Actinidia* DRM1 candidates is indicated by the presence of two distinct families (Type I and Type II) characterised by alternative start methionines, potential phosphorylation sites, and either the absence or presence of a putative PEST motif. Identification of phosphorylated DRM1 peptide fragments in poplar [35] have been identified, but their biological significance remains unclear. In the poplar DRM1 homologue (Ptr_DRM2), three phosphorylation isoforms have identified associated with dormant buds [35]. Of these three phosphorylated residues, namely T^61^, T^63^ and T^70^, two are conserved in the *Actinidia* DRM1 Type I family members corresponding to T^61^ and T^63^ at positions T^55^ and T^57^, respectively. Additional neighbouring residues, such as P, have also been shown to be found in regions involved in transient protein-protein interactions, known to be regulated by phosphorylation and have been proposed to have a role in the prevention of protein aggregation [37]. The presence of methionine in the immediate vicinity of the predicted phosphorylation sites may also suggest a potential additional degree of protein regulation via methionine oxidisation, which has recently been implicated in a stress-induced ROS-mediated signalling cascade in plants [36]. Only *Actinidia* DRM1 Type II family members exhibit a putative PEST motif spanning potential phosphorylation residues (Figure S7). It has been suggested that such PEST motifs are associated with short-lived proteins and result in a reduction in the half-life of the mature proteins, which subsequently become targeted for proteolytic degradation [39].

Additional phylogenetic analysis of the full length *Actinidia* DRM1 deduced protein sequences with bioinformatically mined DRM1 candidates from other plant species indicated that the entire *Actinidia* DRM1 family aligns with the major DRM1 Clade 1 only, with no representatives associated with Clade 2a or 2b. Clade 1 members also include the well-characterised plant DRM1 family members Psa_DRM1, Ath_DRM1 and Rps_ARP. Clade 2a members possess a conserved Domain 1 only, whilst the extended Clade 2b members possess a conserved Domain 1 and a partial conservation of Domain 2. Splice variation is evident, particularly with regards to Ade_DRM1_ID and IG. This pattern of intron retention has also been observed with Ath_DRM1 [40] and *Manihot esculenta* (unpublished data). Alternative splicing is a common phenomenon in plant species, with approximately 7–10% of *Arabidopsis thaliana* transcripts generated as a consequence of shuffled exons, alternative 5′ or 3′ splice sites and different transcript termini with retained introns representing approximately 2–3% of this total figure. The majority of retained introns observed (65%) are the consequence of being either part of open reading frames, present in the UTR region, or present as the last intron in the transcript. This suggests that the presence of such retained introns will not evoke the non-sense-mediated decay process [40]. Biotic and abiotic stresses have previously been shown to affect the patterns of gene splicing in plants, although the precise mechanism remains unknown. It is feasible that the generation of an alternatively spliced transcript may lead to transcript stability or serve to modify the gene's biological function [40].

Both *Actinidia* DRM1 families (I and II) bioinformatically align more closely with Ath_DRM1 rather than the closely related Ath_DRM2, with no putative *Actinidia* homologues for Ath_DRM2 identified, despite reiterative blast analyses. This observation is also borne out in apple (*Malus* x *domestica* ‘Golden Delicious’) and avocado (*Persea americana*). It is feasible that the Ath_DRM2 gene may be the consequence of an ancient paleopolyploidy event independent to the lineage of kiwifruit, or that Ath_DRM2 may be the result of one of the very ancient doubling events leading to the common ancestral eudicot genome, which was subsequently lost in the kiwifruit specific lineage [41].

Intrinsically disordered proteins (IDPs) are surprisingly common in biological systems and comprise approximately 25% to 30% of eukaryotic proteomes, with additional data suggesting that over 50% of eukaryotic proteins and 70% of signalling associated proteins have regions of long disorder [33]. Amino acid composition analyses of the *Actinidia* DRM1 protein family have clearly demonstrated that the members of this family are enriched in major disorder-promoting residues and depleted in major order-promoting residues; in a manner typical for structurally characterized IDPs [42]. Advanced predictive programs aimed at determining the presence of known protein structural motifs in this family failed to identify any such entities, further supporting the observation that this family is indeed disordered at the structural level. The flexibility offered by such disorder may be significant in signalling events, particularly via the function of α-MoRFs. The DRM1 protein family, although encoding for relatively small proteins (<20 KDa), have been predicted to possess up to three such motifs. Binding via a α-MoRF is usually accompanied by a disorder to order transition, as demonstrated recently with the Della transcription factor [43]. Because of their extensive disorder and flexibility, it is suggested that the DRM1 protein family might be able to bind to multiple partners, thereby assuming the role of hub proteins in signalling cascades. On the whole, the biological role of IDPs, particularly in the plant kingdom, remains undetermined and indeed there is a paucity of knowledge surrounding such IDPs. Nevertheless, with the increasing awareness of such proteins, on-going attempts to classify IDPs into biological functions have been undertaken [29]. Based on Biological Processes GO terms and the distribution of the protein in a CH-CDF plot, it has been demonstrated that IDPs do indeed have different subtypes and that the different subtypes may have different biological functions. The *Actinidia* DRM1 protein family has been shown to segregate predominantly into Q4 (quadrant representing proteins predicted to be intrinsically disordered by both CH- and CDF-plot analyses), with only the intron-retaining Ade-DRM1_IG predicted to lie within Q3 (quadrant representing proteins predicted to be intrinsically disordered by CDF-plot and compact by CH-plot analyses) (Figure 6), indicative of the intrinsic disorder nature of this protein family. For proteins in Q4, it has been proposed that they are predominantly mitosis related, such as the G1/S transition of the mitotic cell cycle and responsiveness to salt stress, whereas proteins located in Q3 are mostly associated with regulation or developmental pathways including, for example, negative regulation of cell differentiation, regulation of cell proliferation, negative regulation of signal transduction and response to heat [29].

Analyses of the expression profiles of Ade_DRM1_1G during the winter period and in the presence of HC clearly indicate an inverse correlation between the presence of the transcript and the growth potential of the bud. The use of HC in several commercial fruit industries has been well documented and has been demonstrated to promote budbreak. Its mode of action however, has not been definitively determined, but is thought to be mediated via a H_2_O_2_-mediated signalling cascade in response to sub-lethal stress and disruption of the mitochondrial electron transport chain (mETC) [13], [22], [44]. Upon the addition of HC to the kiwifruit buds, a rapid down-regulation of the Ade_DRM1_1G transcript is evident, followed by a concomitant increase in the Ade_CDKB transcript and a decrease in time to visible physiological budbreak. Of note is the apparent loss of Ade_DRM1_IG transcript in basal bud tissue at days +34 and +42 following HC application. Kiwifruit vines display a strong apical dominant growth form (paradormancy) thought to be mediated via auxin. These data suggest that although DRM1 expression is correlated with a loss of ecodormancy and an enhanced potential to break bud, it is highly likely that the effects of the auxin-mediated paradormancy response dominate the growth habit of the basal bud. The DRM1 family in other plant species has also been shown to be responsive to cold and salt treatments [45], pathogen attack [46] and fungal infection [19], as well as responding to loss of apical dominance via decapitation in arabidopsis [14] and pea [16] in all instances demonstrating an inverse correlation with growth potential. Environmental stresses, such as those previously described, greatly affect the metabolism and growth of the plant and in order to survive plants have developed a complex signalling network that exploits a variety of different growth regulators. One of the common responses to both biotic and abiotic stresses is the formation of reactive oxygen species (ROS). Excessive and rapid accumulation of cellular ROS results in oxidative stress with associated cell damage and/or cell death. However, there is also a growing body of evidence that suggests that low cellular concentrations of ROS, particularly H_2_O_2_, are also an integral aspect of cell signalling and redox sensing mechanisms important for the survival of the plant during environmental stress [44], [47], and may even function as developmental signals associated with several aspects of plant growth [13], [48]. Several oxidative stress and hypoxia-related genes, such as glutathionine S-transferase (GST), sucrose synthase (SuSy), pyruvate decarboxylase (PDC), alcohol dehydrogenase (ADH) and SNF-like protein kinase (GDBRPK), have previously been shown to be upregulated in both kiwifruit [22] and grape [13] dormant buds following exposure to HC. A direct physical measurement of H_2_O_2_ in HC-challenged dormant grape was shown to increase, thus supporting a potential biological role for oxidative perturbation in the early release of bud dormancy [13]. Taken together, these data suggest that the release of HC-mediated bud dormancy in kiwifruit, similar to other species, is likely to be orchestrated via a H_2_O_2_-regulated signalling cascade, with the DRM1 transcript correlating tightly with the growth potential of the bud. The possibility that phosphorylation of the DRM1 protein may be directly modified via H_2_O_2_-mediated methionine oxidation is attractive, but remains to be determined experimentally. Attempts to obtain or generate null mutant lines in *Arabidopsis thaliana* have failed (G. Rae *et al.,* unpublished) suggesting that loss of this highly conserved gene may be lethal to the plant as a whole. Based on these observations, it is apparent that the intrinsically disordered DRM1 protein is not simply a convenient gene marker for paradormancy release [14], [16], but may play a role in an ROS-mediated growth response.

## Supporting Information

Figure S1 **qRT-PCR primer sequence and amplicon size (bp).**
(TIF)

Figure S2 **Effect of HC application upon Ade_Actin transcript in kiwifruit buds.** A: Analysis of Ade_Actin (FG470439) transcript profile in the absence (NHC) or presence (HC) of Hicane from days 0 to 90 post Hicane application (Budbreak 2000) and B: Analysis of Ade_Actin (FG470439) transcript profile in the absence (NHC) or presence (HC) of Hicane from days 0 to 41 post Hicane application (Budbreak 2004).
(TIF)

Figure S3 **Conceptual translation and GenBank ID of putative full length DRM1 candidates from available plant species.** * These two candidates are from different species, but share the same abbreviated genus/species moniker.
(DOCX)

Figure S4 **Sequence annotation and bioinformatic statistics of putative** ***Actinidia*** **(sp) DRM1 homologue ORFs.** (A) Sequence Annotation and (B): Bioinformatic Statistics of *Actinidia deliciosa* (FG468621): Ade_DRM1_IA; *Actinidia deliciosa* ^1^ (FG458205): Ade_DRM1_ID; *Actinidia deliciosa* (FG412327): Ade_DRM1_IE; *Actinidia deliciosa* ^1^ (FG497274); Ade_DRM1_IG; *Actinidia deliciosa* ^1^ (FG449491): Ade_DRM1_IIA.1; *Actinidia deliciosa* ^1^ (FG439737): Ade_DRM1_IIA.3; *Actinidia deliciosa* ^1^ (FG494950): Ade_DRM1_IIA.4; *Actinidia deliciosa* (FG467047): Ade_DRM1_IID.1; *Actinidia deliciosa* (FG439480): Ade_DRM1_IID.2; *Actinidia deliciosa* (FG43983): Ade_DRM1_IID.3. *Actinidia deliciosa^1^*: non-redundant contiguous sequences contain both *Actinidia deliciosa* and *Actinidia chinensis* expressed sequence tag (EST) sequences.
(TIF)

Figure S5 **Alignment and sequence annotation of putative full length cDNA** ***Actinidia*** **(sp) DRM1 homologues Ade_DRM1_IA; Ade_DRM1_ID; Ade_DRM1_IE; Ade_DRM1_IG and Ach_DRM1 gDNA.** *Actinidia deliciosa^1^*: non-redundant contiguous sequences contain both *Actinidia deliciosa* and *Actinidia chinensis* expressed sequence tag (EST) sequences; ^§^for ease of analysis, diploid *Actinidia chinensis* gDNA was isolated and used for sequence comparison.
(TIF)

Figure S6 **Multiple sequence alignment of plant DRM1 family conceptual proteins.** *Actinidia deliciosa* (FG468621): Ade_DRM1_IA; *Actinidia deliciosa^1^* (FG458205): Ade_DRM1_ID; *Actinidia deliciosa* (FG412327): Ade_DRM1_IE; *Actinidia deliciosa^1^* (FG497274); Ade_DRM1_IG; *Actinidia deliciosa^1^* (FG449491): Ade_DRM1_IIA.1; *Actinidia deliciosa^1^* (FG494950): Ade_DRM1_IIA.4; *Actinidia deliciosa* (FG467047): Ade_DRM1_IID.1; *Arabidopsis thaliana* (At1g28330; NP_001154378): Ath_DRM1; *Arabidopsis thaliana* (At2g33830; NP_850220): Ath_DRM2; *Arabidopsis thaliana* (At1g54070; NP_175809): Ath_DRM3; *Arabidopsis thaliana* (At1g56220; NP_849820): Ath_DRM4; *Arabidopsis thaliana* (At5g44300; NP_199243): Ath_DRM5; *Arachis hypogaea* (AAZ20292): Ahy_DRM1; *Brassica oleracea* (AAL67436): Bol_DRM1; *Brassica rapa* (ACQ90305): Bra_DRM1; *Capsicum annum* (Q56UQ6): Can_ARP1; *Citrullus lanatus* (BAI52956): Cla_DRM1^a^; *Codonopsis lanceolata* (AAW02792): Cla_DRM1^b^; *Elaeagnus umbellate* (AAC62104): Eum_ARP1^†^; *Fragaria* x *ananassa* (Q05349): Fan_λSAR5^†^; *Glycine max* (ACU23540): Gma_DRM1; *Glycyrrhiza uralensis* (ABR15095): Gur_DRM1; *Malus* x *domestica* (AAA71994): Mdo_AP1^†^; *Malus* x *domestica* (AAK25768): Mdo_AP1L^†^; *Manihot esculenta* (AAX84677): Mes_DRM1; *Medicago truncatula* (ACJ83865): Mtr_DRM1; *Mirabilis jalapa* (AAN16890): Mja_DRM1; *Nicotiana tabacum* (AAO21304): Nta_ARPL1^†^; *Nicotiana tabacum* (AAS76635): Nta_ARP1^†^; *Nicotiana tabacum* (ABY16785): Nta_ARP2^†^; *Oryza sativa* Japonica group (ABA95234): Osa_DRM1; *Oryza sativa* Japonica group (ABF95871): Osa_DRM2; *Oryza sativa* Japonica group (NP_001061955): Osa_DRM4; *Oryza sativa* Japonica group (NP_001063265): Osa_DRM5; *Oryza sativa* (AAL78369): Osa_DRM7; *Oryza sativa* Indica group (EEC83671): Osa_DRM8; *Paeonia suffruticosa* (ABW74471): Psu_ARP^†^; *Physcomitrella patens* subsp. Patens (XP_001755658): Ppa_DRM1; *Physcomitrella patens* subsp. Patens (XP_001780946): Ppa_DRM2; *Physcomitrella patens* subsp. Patens (XP_001781096): Ppa_DRM3; *Picea sitchensis* (ABK21467): Psi_DRM1; *Picea sitchensis* (ABK22604): Psi_DRM2; *Picea sitchensis* (ABK23285): Psi_DRM3; *Picea sitchensis* (ABK23718): Psi_DRM4; *Picea sitchensis* (ACN41230): Psi_DRM5; *Pisum sativum* (AAB84193): Psa_DRM1;*Pisum sativum* (AAM62421): Psa_DRM3; *Pisum sativum* (AAM62422):Psa_DRM4; *Populus trichocarpa* (XP_002304241): Ptr_DRM1; *Populus trichocarpa* (XP_002305123): Ptr_DRM2; *Populus trichocarpa* (XP_002319507): Ptr_DRM3; *Populus trichocarpa* (XP_002330171): Ptr_DRM4; *Prunus armeniaca* (AAB88876): Par_DRM1; *Pyrus pyrifolia* (ACJ68422): Ppy_DRM1; *Pyrus pyrifolia* (ACN97421): Ppy_DRM2; *Ricinus communis* (XP_002509446): Rco_DRM1; *Ricinus communis* (XP_002512449): Rco_DRM2; *Ricinus communis* (XP_002529178): Rco_DRM3; *Robinia pseudoacacia* (AAG33924): Rps_ARP^†^; *Sesbania drummondii* (ABQ44282): Sdr_DRM1; *Solanum lycopersicum* (ABH07900): Sly_DRM1; *Solanum tuberosum* (ABA40468): Stu_DRM1; *Solanum virginianum* (AAS75891): Svi_DRM1; *Sorghum bicolor* (XP_002460264): Sbi_DRM1; *Sorghum bicolor* (XP_002465296): Sbi_DRM2; *Vitis vinifera* (XP_002269240): Vvi_DRM1; *Vitis vinifera* (XP_002279836): Vvi_DRM2; *Vitis vinifera* (XP_002283180): Vvi_DRM3; *Zea mays* (ACG37064): Zma_DRM1; *Zea mays* (ACG39507): Zma_DRM2; *Zea mays* (NP_001130689): Zma_DRM3; *Zea mays* (NP_001150581): Zma_DRM4. *Note: All conceptual translation sequences assigned an arbitrary DRM1 nomenclature except where previously described in the literature (^†^) (See Figure S2 for predicted protein sequence). Actinidia deliciosa^1^*: non-redundant contiguous sequences contain both *Actinidia deliciosa* and *Actinidia chinensis* expressed sequence tag (EST) sequences.
(TIF)

Figure S7 **Putative α-MoRF; phosphorylation sites and PEST motif annotation of conceptually translated putative full length DRM1 candidates from kiwifruit.**
(TIF)
