# Supplementary figures and images for: Actinidia DRM1 - An Intrinsically Disordered Protein Whose mRNA Expression Is Inversely Correlated with Spring Budbreak in Kiwifruit
Source: PLoS One. 2013 Mar 13;8(3):e57354. doi: 10.1371/journal.pone.0057354 (PMC3596386; doi:10.1371/journal.pone.0057354)

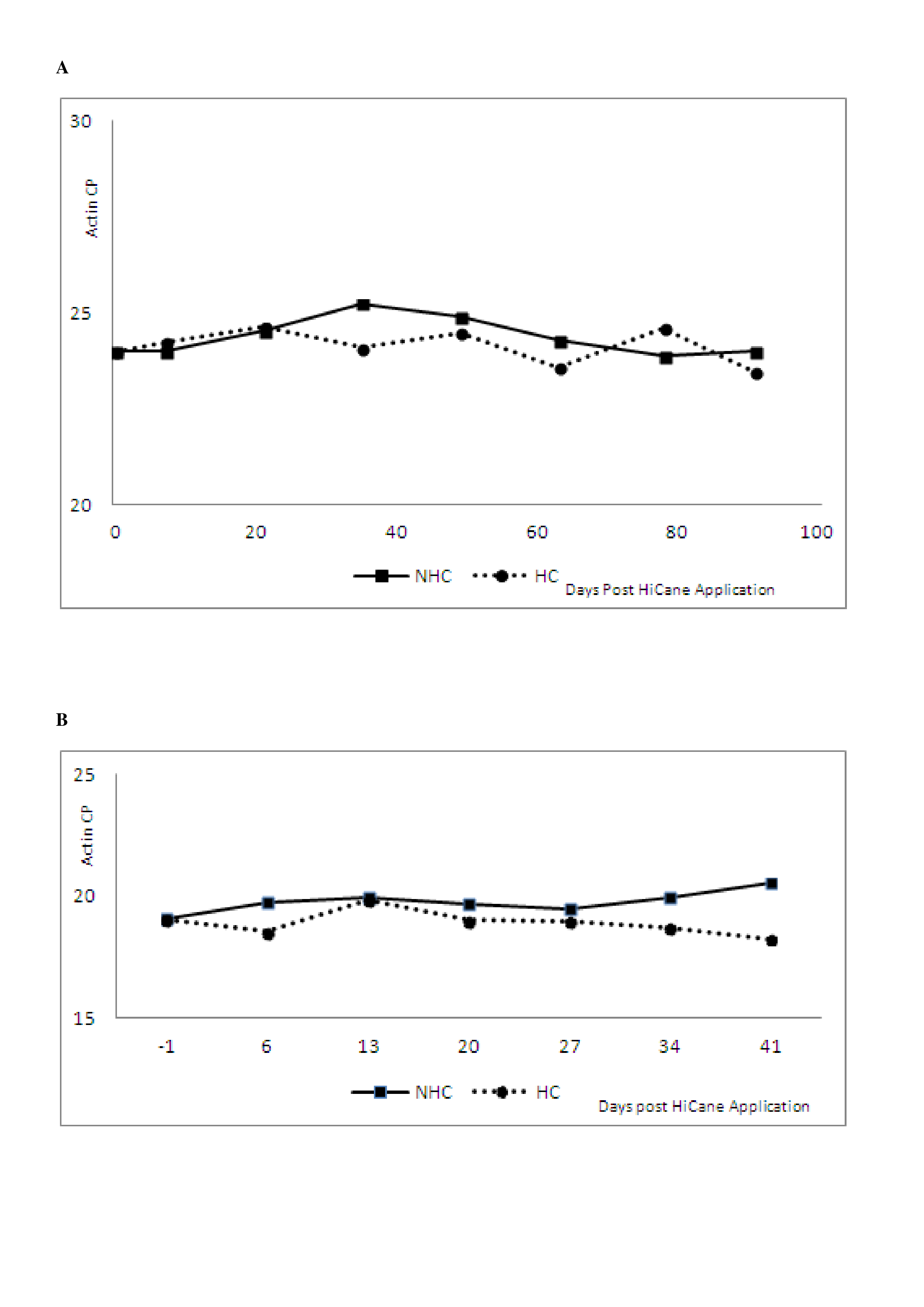

Supplement: Figure S2 — Effect of HC application upon Ade_Actin transcript in kiwifruit buds. A: Analysis of Ade_Actin (FG470439) transcript profile in the absence (NHC) or presence (HC) of Hicane from days 0 to 90 post Hicane application (Budbreak 2000) and B: Analysis of Ade_Actin (FG470439) transcript profile in the absence (NHC) or presence (HC) of Hicane from days 0 to 41 post Hicane application (Budbreak 2004). (TIF) [file pone.0057354.s002.tif]

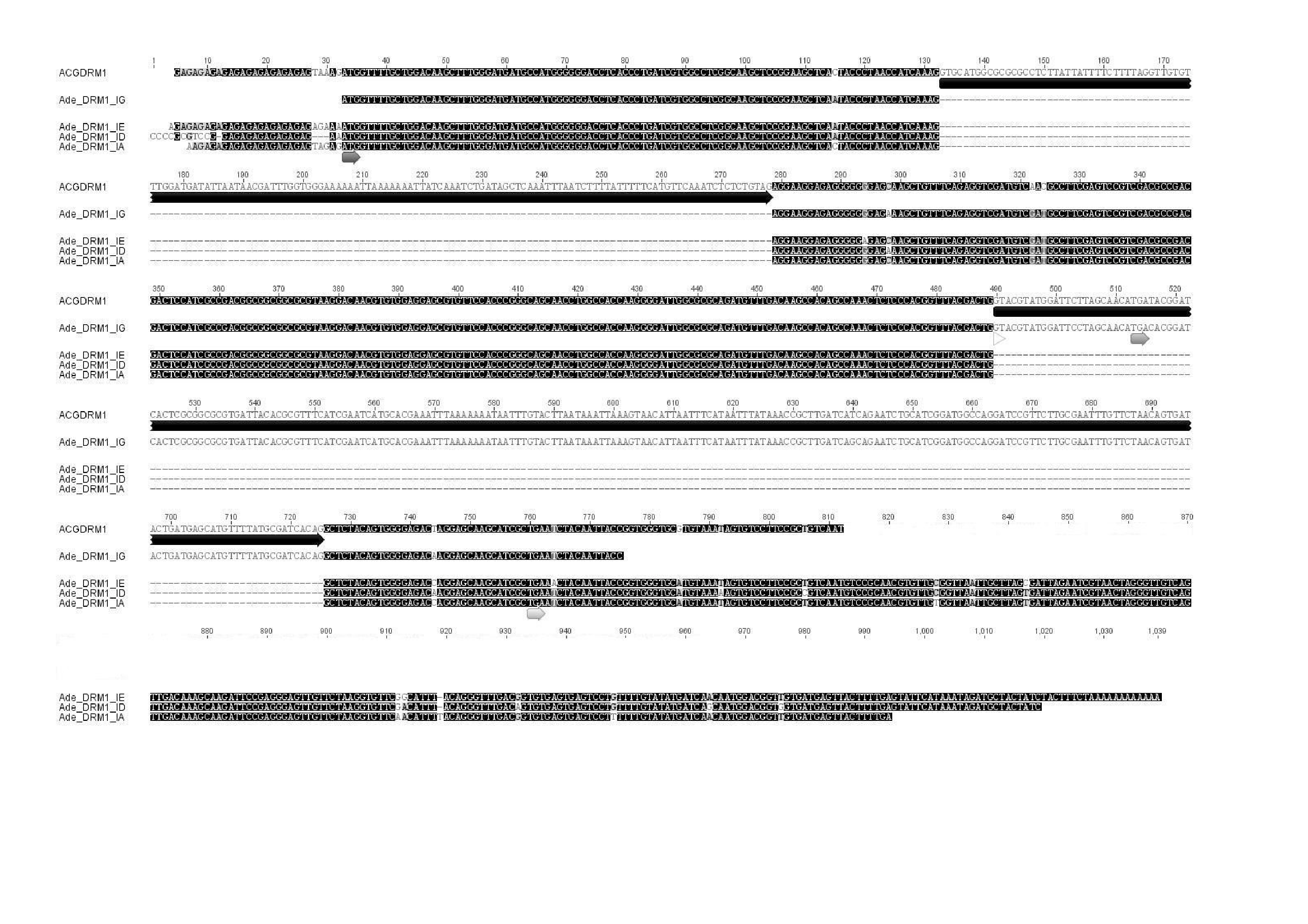

Supplement: Figure S5 — Alignment and sequence annotation of putative full length cDNA Actinidia (sp) DRM1 homologues Ade_DRM1_IA; Ade_DRM1_ID; Ade_DRM1_IE; Ade_DRM1_IG and Ach_DRM1 gDNA. Actinidia deliciosa1: non-redundant contiguous sequences contain both Actinidia deliciosa and Actinidia chinensis expressed sequence tag (EST) sequences; §for ease of analysis, diploid Actinidia chinensis gDNA was isolated and used for sequence comparison. (TIF) [file pone.0057354.s005.tif]

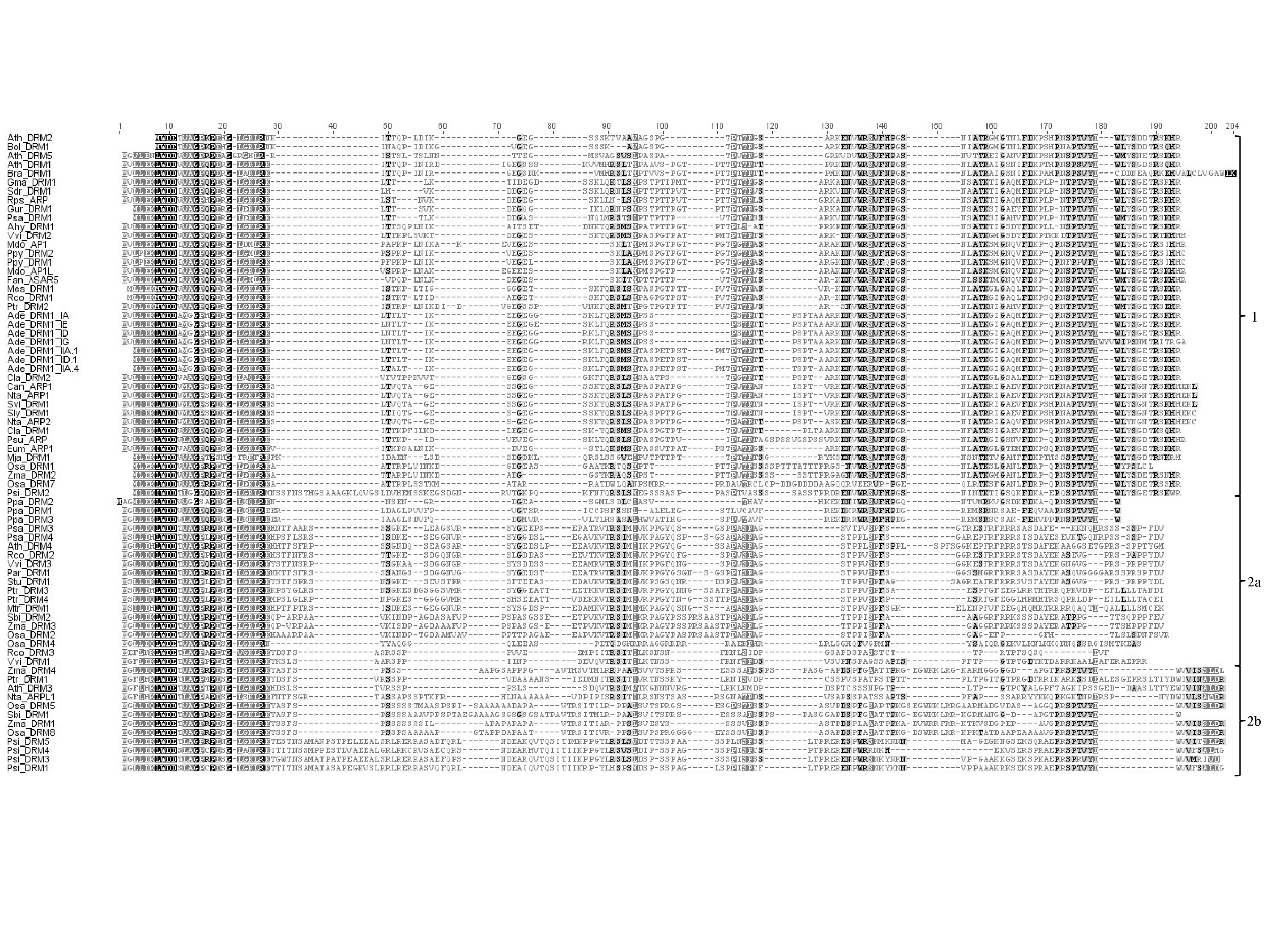

Supplement: Figure S6 — Multiple sequence alignment of plant DRM1 family conceptual proteins. Actinidia deliciosa (FG468621): Ade_DRM1_IA; Actinidia deliciosa1 (FG458205): Ade_DRM1_ID; Actinidia deliciosa (FG412327): Ade_DRM1_IE; Actinidia deliciosa1 (FG497274); Ade_DRM1_IG; Actinidia deliciosa1 (FG449491): Ade_DRM1_IIA.1; Actinidia deliciosa1 (FG494950): Ade_DRM1_IIA.4; Actinidia deliciosa (FG467047): Ade_DRM1_IID.1; Arabidopsis thaliana (At1g28330; NP_001154378): Ath_DRM1; Arabidopsis thaliana (At2g33830; NP_850220): Ath_DRM2; Arabidopsis thaliana (At1g54070; NP_175809): Ath_DRM3; Arabidopsis thaliana (At1g56220; NP_849820): Ath_DRM4; Arabidopsis thaliana (At5g44300; NP_199243): Ath_DRM5; Arachis hypogaea (AAZ20292): Ahy_DRM1; Brassica oleracea (AAL67436): Bol_DRM1; Brassica rapa (ACQ90305): Bra_DRM1; Capsicum annum (Q56UQ6): Can_ARP1; Citrullus lanatus (BAI52956): Cla_DRM1a; Codonopsis lanceolata (AAW02792): Cla_DRM1b; Elaeagnus umbellate (AAC62104): Eum_ARP1†; Fragaria x ananassa (Q05349): Fan_λSAR5†; Glycine max (ACU23540): Gma_DRM1; Glycyrrhiza uralensis (ABR15095): Gur_DRM1; Malus x domestica (AAA71994): Mdo_AP1†; Malus x domestica (AAK25768): Mdo_AP1L†; Manihot esculenta (AAX84677): Mes_DRM1; Medicago truncatula (ACJ83865): Mtr_DRM1; Mirabilis jalapa (AAN16890): Mja_DRM1; Nicotiana tabacum (AAO21304): Nta_ARPL1†; Nicotiana tabacum (AAS76635): Nta_ARP1†; Nicotiana tabacum (ABY16785): Nta_ARP2†; Oryza sativa Japonica group (ABA95234): Osa_DRM1; Oryza sativa Japonica group (ABF95871): Osa_DRM2; Oryza sativa Japonica group (NP_001061955): Osa_DRM4; Oryza sativa Japonica group (NP_001063265): Osa_DRM5; Oryza sativa (AAL78369): Osa_DRM7; Oryza sativa Indica group (EEC83671): Osa_DRM8; Paeonia suffruticosa (ABW74471): Psu_ARP†; Physcomitrella patens subsp. Patens (XP_001755658): Ppa_DRM1; Physcomitrella patens subsp. Patens (XP_001780946): Ppa_DRM2; Physcomitrella patens subsp. Patens (XP_001781096): Ppa_DRM3; Picea sitchensis (ABK21467): Psi_DRM1; Picea sitchensis (ABK22604): Psi_DRM2; P [file pone.0057354.s006.tif]

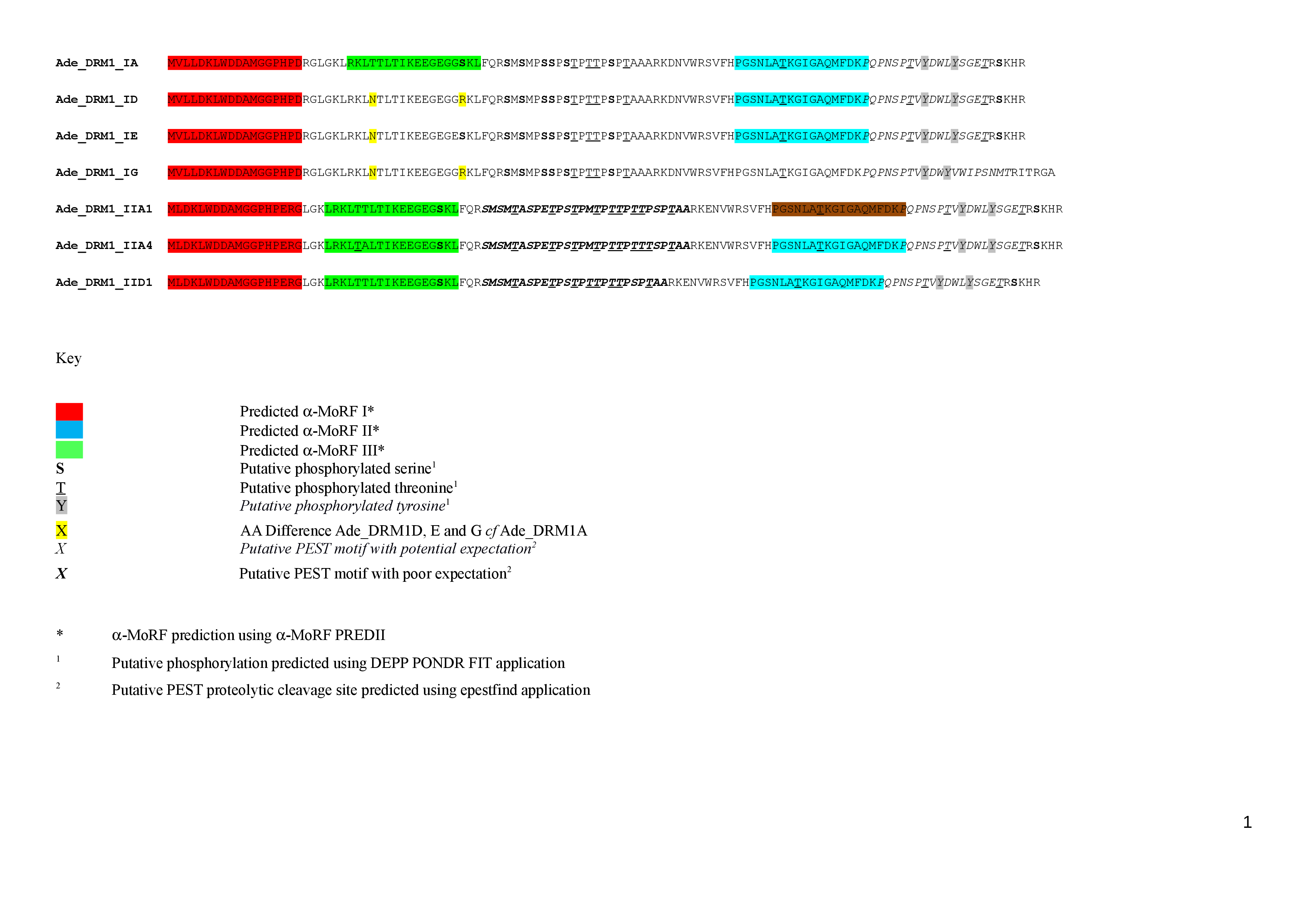

Supplement: Figure S7 — Putative α-MoRF; phosphorylation sites and PEST motif annotation of conceptually translated putative full length DRM1 candidates from kiwifruit. (TIF) [file pone.0057354.s007.tif]
