# Supplementary material for: Actinidia DRM1 - An Intrinsically Disordered Protein Whose mRNA Expression Is Inversely Correlated with Spring Budbreak in Kiwifruit
Source: PLoS One. 2013 Mar 13;8(3):e57354. doi: 10.1371/journal.pone.0057354 (PMC3596386; doi:10.1371/journal.pone.0057354)
Supplement: Figure S3 — Conceptual translation and GenBank ID of putative full length DRM1 candidates from available plant species. * These two candidates are from different species, but share the same abbreviated genus/species moniker. (DOCX) [file pone.0057354.s003.docx]

| **Species** | **Protein Identifier** | **Abbreviated ID** | **Conceptually Translated Protein Sequence** | **References** | **Size**  **(aa)** |
| --- | --- | --- | --- | --- | --- |
| *Actinidia deliciosa* | FG468621 | Ade_DRM1_IA | MVLLDKLWDDAMGGPHPDRGLGKLRKLTTLTIKEEGEGGSKLFQRSMSMPSSPSTPTTPSPTAAARKDNVWRSVFHPGSNLATKGIGAQMFDKPQPNSPTVYDWLYSGETRSKHR* |  | 115 |
| *Actinidia deliciosa* | FG458205 | Ade_DRM1_ID | MVLLDKLWDDAMGGPHPDRGLGKLRKLNTLTIKEEGEGGRKLFQRSMSMPSSPSTPTTPSPTAAARKDNVWRSVFHPGSNLATKGIGAQMFDKPQPNSPTVYDWLYSGETRSKHR* |  | 115 |
| *Actinidia deliciosa* | FG412327 | Ade_DRM1_IE | MVLLDKLWDDAMGGPHPDRGLGKLRKLNTLTIKEEGEGESKLFQRSMSMPSSPSTPTTPSPTAAARKDNVWRSVFHPGSNLATKGIGAQMFDKPQPNSPTVYDWLYSGETRSKHR* |  | 115 |
| *Actinidia deliciosa* | FG497274 | Ade_DRM1_IG | MVLLDKLWDDAMGGPHPDRGLGKLRKLNTLTIKEEGEGGRKLFQRSMSMPSSPSTPTTPSPTAAARKDNVWRSVFHPGSNLATKGIGAQMFDKPQPNSPTVYDWYVWIPSNMTRITRGA* |  | 119 |
| *Actinidia deliciosa* | FG449491 | Ade_DRM1_IIA.1 | MLDKLWDDAMGGPHPERGLGKLRKLTTLTIKEEGEGSKLFQRSMSMTASPETPSTPMTPTTPTTPSPTAARKENVWRSVFHPGSNLATKGIGAQMFDKPQPNSPTVYDWLYSGETRSKHR* |  | 120 |
| *Actinidia deliciosa* | FG494950 | Ade_DRM1_IIA.4 | MLDKLWDDAMGGPHPERGLGKLRKLTALTIKEEGEGSKLFQRSMSMTASPETPSTPMTPTTPTTTSPTAARKENVWRSVFHPGSNLATKGIGAQMFDKPQPNSPTVYDWLYSGETRSKHR* |  | 120 |
| *Actinidia deliciosa* | FG467047 | Ade_DRM1_IID.1 | MLDKLWDDAMGGPHPERGLGKLRKLTTLTIKEEGEGSKLFQRSMSMTASPETPSTPTTPTTPSPTAARKENVWRSVFHPGSNLATKGIGAQMFDKPQPNSPTVYDWLYSGETRSKHR* |  | 117 |
| *Arabidopsis thaliana* | NP_001154378 (At1g28330) | Ath_DRM1 | MVLLEKLWDDVVAGPQPDRGLGRLRKITTQPINIRDIGEGSSSKVVMHRSLTMPAAVSPGTPTTPTTPTTPRKDNVWRSVFNPGSNLATRAIGSNIFDKPTHPNSPSVYDWLYSGDSRSQHR* |  | 122 |
| *Arabidopsis thaliana* | NP_850220 (At2g33830) | Ath_DRM2 | MWDETVAGPKPEHGLGRLRNKITTQPLDIKGVGEGSSSKTVAAVAGSPGTPTTPGSARKENVWRSVFHPGSNIATRGMGTNLFDKPSHPNSPTVYDWLYSDDTRSKHR* |  | 108 |
| *Arabidopsis thaliana* | NP_175809 (At1g54070) | Ath_DRM3 | MGFLHKLWDETVAGPTPDNGLGKLRKHDSLSTVRSSPPSLSSDQVTRSIMVTKGNNNVRGLRKLKMDPDSPTCSSSNPGTPLTPGTPCYALGPFTAGKIPSSGEDDAASLTTYEWIVINALDR* |  | 123 |
| *Arabidopsis thaliana* | NP_849820 (At1g56220) | Ath_DRM4 | MGLLDHLWDDTVAGPRPENGLGKLRKHHTFSFRPSSGNDQSEAGSARSYGEDSLPEEAVKVTRSIMIIKPPGYQGSSAPASPAGSTPPLSPFSPPLSPFSANAGGKEPFRFRRRSTSDAFEKAAGGSETGPRSSPPTYGM* |  | 140 |
| *Arabidopsis thaliana* | NP_199243 (At5g44300) | Ath_DRM5 | MGVLENLWDDVVAGPRPEAGGRGHLRRISTSLTSLNNTTEGMSVAGSVSLPASPATPVTPGSGRKVDVWRSVFHPASNVTTREIGANVFDKPSHPNSPTVYDWMYSNETRSKHR* |  | 114 |
| *Arachis hypogaea* | AAZ20292 | Ahy_DRM1 | MVLLEKLWDDVVAGPQPDRGLGKLRKITTSQPLNIKAITSETDNKYQRSMSMPATPTTPGTPTTPLSATPRKPDNVWRSVFHPGSNSATKTIGSDYFDKPLLNSPTVYDWLYSGETRSKHR* |  | 121 |
| *Brassica oleracea* | AAL67436 | Bol_DRM1 | MWDETVAGPKPEHGLGRLRNKINAQPIDIKGVGEGSSSKAVAGSPGTPTTPGSARKENVWRSVFHPGSNIATRGMGTNLFDKPSHPNAPTVYDWLYSDDTRSQHR* |  | 105 |
| *Brassica rapa* | ACQ90305 | Bra_DRM1 | MVLLDKLWDDVVAGPQPDRGLARLRKITTQPINIRGEGSNKVMHRSLTMPTVVSPGTPTTPTTPTTPHKDNVWRSVFNPGSNLATRAIGSNIFDKPAHPNSPSVYDCDDNEAQRKEHVALCLVGAWIK* |  | 128 |
| *Capsicum annuum* | Q56UQ6 | Can_ARP1 | MVLIDKLWDDVMAGPSPDKGLGKLRKSLTVQTAGESSGEGSSKYQRSLSMPASPATPGTP  VTPANISPTVRKENVWRSVFHPGSNLATKRIGAEVFDKPSHPNAPTVYDWLYSGNTRSKH  HEKL* |  | 124 |
| *Citrullus lanatus* | BAI52956 | Cla_DRM1^a^* | MVLLEKLWDDVVAGPQPDRGLGKLRKITTKPFILKDLEGEGSKYQRSMSMPASPGTPTTPVTPTTPLTAARKDNVWRSVFNPGRNFATKSIGAEVFDKPQPNSPTVYDWLYSGDTKSQHR* |  | 120 |
| *Codonopsis lanceolata* | AAW02792 | Cla_DRM1^b^* | MVLIDKLWDDVAAGPQPDHGLAQLRKVFVTPPKVVTGEGSGGKFFQRSLSMSAATPSTPGTPTTPSPTARKDNVWRSVFNPGSNLATKGLGSALFDKPEPNSPTVYDWLYSGETRSKHR* |  | 119 |
| *Elaeagnus umbellata* | AAC62104 | Eum_ARP1 | MVLLDKIWDDVAAGPQPESGLGRLRKVITKPSALNIKDVEGSTLQKSMSMPASSVTPATPSTPATPGSARKENVWRSVFNPGSNLATRGLGTEMFDKPSQPNSPTVYDWLYSGETRSKHR* | [1] | 120 |
| *Fragaria* x *ananassa* | Q05349 | Fan_λSAR5 | MVLLDKLWDDIVAGPQPERGLGMLRKVPQPLNLKDEGESSKITMPTTPTTPVTPTTPISARKDNVWRSVFHPGSNLSSKTMGNQVFDSPQPNSPTVYDWMYSGETRSKHHR* | [2] | 111 |
| *Glycine max* | ACU23540 | Gma_DRM1 | MVLLEKLWDDVVAGPQPERGLGKLRKLTTLKTIDEGDSSKLQKTLSMPSTPTIPMTPTTPTTPGSARKADNVWRSVFHPGSNSATKTIGAQMFDKPLPNTPTVYDWLYSGETRSRHR* |  | 117 |
| *Glycyrrhiza uralensis* | ABR15095 | Gur_DRM1 | MLEKLWDDVVAGPQPERGLERLRKLTTSVKDEGQGIKLQRNPSMPSTPTTPGTPTTPTTPGSARKADNVWRSVFNPGSNSATKSIGAEYFDKPLPNTPTVYDWLYSGDTRSKHR* |  | 114 |
| *Malus* x *domestica* | AAA71994 | Mdo_AP1 | MVLLEKLWDDIVAGPQPERGLDMLRRPAPKPLNIKAKEVEGESSKLTMPMSPGTPGTPGTPGTPASARAKDNVWRSVFHPGSNLATKSMGNQVFDKPQPNSPTVYDWLYSGETRSIHHR* | [3] | 119 |
| *Malus* x *domestica* | AAK25768 | Mdo_AP1L | MVLLEKLWDDIVAGPQPERGLGRLRKVSPRPLNAKEGEEESSKLAMPMSPGTPGTPGTPVSARAKDNVWRSVFHPGSNLASKSMGNQVFDKPQPNSPTVYDWLYSGETRSKHHR* |  | 114 |
| *Manihot esculenta* | AAX84677 | Mes_DRM1 | MLLDKMWDDVVAGPQPDRGLGKLRKISTKPLTIGGGGETSKFQRSISMPASPGTPTTPVTPTTPASVRKDNVWRSVFHPGSNLATKGLGAQLFDKPQPNSPTVYDWLYSGETRSKHR* |  | 117 |
| *Medicago truncatula* | ACJ83865 | Mtr_DRM1 | MSILDHLWDDTVAGPRPENGLGKLRKHPTFPTRSISDKESGEGGNVRSYSGDSPEDAMKVTRSIMIMKPAGYQSNGSAPASPAGSTPPVSPFSGKELENPFVFEEGQHQMRTRRRRQAQTDQALLLLSMCEK* |  | 132 |
| *Mirabilis jalapa* | AAN16890 | Mja_DRM1 | MLDKLWDDVVAGPTPSHGFRKFRRPKIDAENLSDSDGDKLQRSLSSGVEIPVTPTTPTTPTTPTSGRYKSENVWRSVFNPGSNSNTKTVGAHFFDKPTHSSSPTVYDWLYSGDTRNKRM* |  | 119 |
| *Nicotiana tabacum* | AAO21304 | Nta_ARPL1 | MGFLHKLWDDTLAGPAPDSGLSKLRKFNTFSGRTASSAPSSPTKFRHLNAAAAAAVDPIPISRSITILRSNSTSASRSGNSPATSSAPTSPFAPSSARRYYKKQPKGKTNRERSPNYDWIVLSAWDR* | [4] | 127 |
| *Nicotiana tabacum* | AAS76635 | Nta_ARP1 | MVLIDKLWDDVMAGPSPDKGLGKLRKSLTVQTAGESSGEGSSKYQRSLSMPASPATPGTPVTPANISPTVRKENVWRSVFHPGSNLATKRIGAEVFDKPSHPNAPTVYDWLYSGNTRSKHHEL* |  | 124 |
| *Nicotiana tabacum* | ABY16785 | Nta_ARP2 | MVLIDKLWDDVMAGPQPDNGLGKLRKSLTVQTGGESGEGSSKYQRSLSMPASPPTPGTPATPTTPSPTASKENVWRSVFHPGSNIATKRIGAQVFDKPSHPNAPTVYDWLYNGNTRSKHHEK* |  | 123 |
| *Oryza sativa* (Japonica group) | ABA95234 | Osa_DRM1 | MLEKLWDDVVAGPRPETGLEKLRKAATTRPLVINKDGDGEASGAAYKRTQSMPTTPTTPVTPSSSSPTTTATTTPRGSNVWRSVFHPGSNLATKSLGANLFDRPQPNSPTVYDWYPSLCL* |  | 120 |
| *Oryza sativa* (Japonica group) | ABF95871 | Osa_DRM2 | MGLLDKLWDDTVAGPRPDTGLGRLRKHAAARPAAVKINDPTGDAAMVAVPPTTPAGAEEAPVKVTRSIMIKRPAGYPASPRSAASTPPASPAGSTPPISPFAGAGEFPGFHTLSLSPNFSVR* |  | 122 |
| *Oryza sativa* (Japonica group) | NP_001061955 | Osa_DRM4 | MGLLDQLWDETVAGPRPDSGLGKLRNYYAQGGQLEEASPETQDGHRRRAGGRRRRRAEKPHRLRLGGHQFVGPMNYSAIQRGEKVLKNLKKQNNQSSRGISMTKEAS* |  | 107 |
| *Oryza sativa* (Japonica group) | NP_001063265 | Osa_DRM5 | MGLLDQLWDDTVAGPRPDSGLGKLRKYASFSPSSSSSTMAASPSPISAAAAAADAPAVTRSITILRPPALSVTSPRGSESGPSTPSSPASVPDSPFGSAPTPKGSEGWKKLRRGAARMADGVDASAGGQPRSPTVYDWVVISSLDR* |  | 146 |
| *Oryza sativa* | AAL78369 | Osa_DRM7 | MLEKLWDDVVAGPRPETGLEKLRKAATTRPLSSTKMATARRATDWLQANPSMRRPRDAVTRCLCPDDGDDDDAAGQQRVEERVPPGEQLRTKSFGANLFDRPQPNSPTVYDWLYSDETRSSHR* |  | 123 |
| *Oryza sativa* Indica group | EEC83671 | Osa_DRM8 | MGLLDQLWDETVAGPRPDSGLGKLRKYSSFSPSSPSAAAAAPGTAPPDAPAATVTRSITIVRPPSLSVPSPRGGGGEYSSSVPSSPASAPDSPFASATTPKGDSWRRLRRKPKTATDAAPEAAAAVGPRSPTVYDWVVISSLDR* |  | 144 |
| *Paeonia suffruticosa* | ABW74471 | Psu_ARP | MVLLDRLWDDVLAGPQPERGLGKLRKITTKPIDVEVEGSKLYQRSLSMPASPGTPVIPLTPTAGSPSSVGSPSSVRKDNVWRSVFNPGSNLATRGIGSNVFDKPQPNSPTVYDWLYSGDTRSKHHR* |  | 126 |
| *Physcomitrella patens* subsp. Patens | XP_001755658 | Ppa_DRM1 | MGLLDKLWDDVVAGPQPEKGLKKLREERLDAGLPVVFPVGTSRICCPSFSSNLALELEGSTLVCAVFREKDKRWRSVFHPDGREMSRNRSAEFEQVAAPNSPTVYDW* |  | 107 |
| *Physcomitrella patens* subsp. Patens | XP_001780946 | Ppa_DRM2 | MAGMLEKLWDDVVGGSAPDKGLKQLRKNNSENGRDGEASGMLSDLCIASVTHAYHKEKDNIWRSVFHPGQNTVMRKVGSDKFDKAQPNSPTVYDW* |  | 95 |
| *Physcomitrella patens* subsp. Patens | XP_001781096 | Ppa_DRM3 | MGLLDKLWDDVLAGPQPDKGLKKLRKERIAAGLSDVFQDGMVRVLYLHSASALHWVATIHGSFPVTAVFREKDRRWRSMFHPEGREMSRSCSAKFEHVPPPNSPTVYDW* |  | 109 |
| *Picea sitchensis* | ABK21467 | Psi_DRM1 | MGLLDKLWDDSLAGPCPDSGLGKLRKTTITNSAMATASAPEGKVSLRRLRERRASVQFQRLNDEAIQVTQSITIIKRPYLHSPSMDSPSSPAGLSPPISPCFLTPRERENPWRSNKYKNNVPPAAAKRKSEKSPRAEPRSPTVYDWVVFSALDG* |  | 154 |
| *Picea sitchensis* | ABK22604 | Psi_DRM2 | MLDKIWDDTLGGPQPDSGLGRLRMNSSFNSTHGSAAAGKLQVGSLDVHEMSSKEGSDGNRVTGKPQKFNFQRSLSMEGSSSASPPASPTVASSSSASSTPRDRENVWRSVFHPGSNINTKTIGSQKFDKAEPQSPTVYDWLYSGETRSKWR* |  | 151 |
| *Picea sitchensis* | ABK23285 | Psi_DRM3 | MGLLDKLWDDTLAGPRPESGLGKLRKTGWTNSAMATPATPEAEEALSRLRERRASAEFQPSNDEARQVTQSITIIKPPGYLRSLSLDSPSSPAGSSSPISPSSLTPRERENPWRSNKYNKNVPGAAKKGSEKSPKAEPRSPRVYDWVVMRIVD* |  | 153 |
| *Picea sitchensis* | ABK23718 | Psi_DRM4 | MGLLDKLWDDSLAGPCPDSGLAKLRKITITNSSMPPESTLVAEEALGRLRKCRVSAECQRSNDEARHVTQIITIIKPPGYLRSVSLDIPSSPAGSSPPISPCSPTPRERENPWRRNKHEKVSERSPRAEPRSPTVYDWVVFSALHG* |  | 146 |
| *Picea sitchensis* | ACN41230 | Psi_DRM5 | MGLLDKLWDETLAGPRPENGLGKLRKTESTNSAMANPSTPELEEALSRLRERRASADFQRLNDEAKQVTQSITIMKPPGYLRSLSVDTTTSSPAASSPLNSPSSLTPRERESPWRSKMKNNNMAGEGKNGSEKSQRAEPRSPTVYDWVVITSLER* |  | 155 |
| *Pisum sativum* | AAB84193 | Psa_DRM1 | MLDKLWDDIVAGPQPERGLEKLRKLTTTLKDDGASNQLMRSTSIPTTPTTPVTPTTPSSARKVDNVWRSVFNPGSNSATKSIGAHVFDKPLPNTPTVYDWMYSGDTRSKHR* |  | 111 |
| *Pisum sativum* | AAM62421 | Psa_DRM3 | MGLLDQLWDDTVAGPRPENGLGKLRKHNTFAARSSSGKELEAGSVRSYGEEPSEPATRVTRSIMIVKPPGYQSGSPPASPAGSVTPVSPFSGTRESFRFRRRSASDAFEKKNQDRSSSSSPFDV* |  | 124 |
| *Pisum sativum* | AAM62422 | Psa_DRM4 | MSLLDHLWDDTVAGPLPENGLGKLRKHPSFLSRSISDKESEGGNVRSYGGDSLEGAVKVTRSIMIVKPAGYQSPSGSAPASPAGSTPPLSPFSGAREPFRFRRRSISDAYESEVKTGQNRPSSSSPFDV* |  | 129 |
| *Populus trichocarpa* | XP_002304241 | Ptr_DRM1 | MGFLHKLWDETLAGPMPDSGLGKLRKYDSFSVRSSPPVDAAAANSIEDMNITRSITIVRTNSSKYLRNISVDPCSSPVSPATPSTPTTPLTPGITGTPRGDFRRIKARKSSDEALESGEPRSLTIYDWIVINALDR* |  | 136 |
| *Populus trichocarpa* | XP_002305123 | Ptr_DRM2 | MVLLDKMWDDVVAGPQPERGLGKLRKISTRPLNIKDIDVGEGSSPVNKFQRSMTMPGTPGTPTTPVTPTTPVSARSNVWRSVFHPGSNLATKNIGAHVFDKPQPNTPTVYDWMYSGETKSEHR* |  | 123 |
| *Populus trichocarpa* | XP_002319507 | Ptr_DRM3 | MSLLDHLWDDTVAGPLPENGLGKLRKKPSYGLRSNSGKESDGSGGSVMRSYGGEATTEETKKVTRSIMIVRPPGYQNNGSSATPPASPAGSTPPVSPFSAESPFGFEEGLRRTHTRRQPRVDPEFLLLLTANDI* |  | 134 |
| *Populus trichocarpa* | XP_002330171 | Ptr_DRM4 | MSLLDQLWDDTVAGPLPESGLGKLRKMPSLGLRPNPGKESGGGGVMRSHSEEATTVDEKRVTRSIMIVRPPGYTNGSSTTPPASPAGSTPPVSPFPESRFGFEGGLHRMHTRSQPRLDPEILLLLTACEI* |  | 130 |
| *Prunus armeniaca* | AAB88876 | Par_DRM1 | MGLLDQLWDDTVAGPRPDIGLGKLRKHKTFSFRSSSANGSSDGGNVGSYGEDSTEEATRVTRSIMIVKPPGYGSGNSGSPPISPAGSTPPVSPFSGGSSMGRFRRRSASDAYEKASQVGGGGARSSPRSPFDV* |  | 133 |
| *Pyrus pyrifolia* | ACJ68422 | Ppy_DRM1 | MVLPEKLWDDIVAGPQPESGLGKLRKPFPKPLNIKVEGELSKLAMPMSPGTPGTPGTPGTPASARGKDNVWRSVFQPGSNLATKSMGNQVFDKPQPNFPPVFDWLYSGETRSIHHC* |  | 116 |
| *Pyrus pyrifolia* | ACN97421 | Ppy_DRM2 | MVLPEKLWDDIVAGPQPERGLGMLRKPSPKPLNIKVEGESSKLAMPMSPGTPGTPGTPGTPASARAKDNVWRSVFHPGSNLATKSMGNQVFDKPQPNSPTVYDWLYSGETRSIHHC* |  | 116 |
| *Ricinus communis* | XP_002509446 | Rco_DRM1 | MLLDKMWDDVVAGPQPDRGLGKLRKISTKTLTIDAEGETSKFQRSLSMPAGPGTPSTPVTPTTPASARKDNVWRSVFHPGSNLATRGIGAQLFDKPSQPNSPTVYDWLYSGETRSKHR* |  | 118 |
| *Ricinus communis* | XP_002512449 | Rco_DRM2 | MGLLDQLWDDTVAGPPPDNGLGKLRKHSTFNFRSTTGKESDGQNGRSLGDDASEEVTKVTRSIMIVKPPGYQFGSPPVSPAGSTPPVSPFSGGRESFRFRRRSTSDAYEKASEVGPRSPAPPYD* |  | 125 |
| *Ricinus communis* | XP_002529178 | Rco_DRM3 | MEFLHKLWDETLAGPAPETGVGKLRKYDSFSASRSSPPVVEEMPIITRSITILKSNSNFKNLSIDPGSAPDSPASSTCTTPRTPFSQSQEVF* |  | 92 |
| *Robinia pseudoacacia* | AAG33924 | Rps_ARP | MVLLEKLWDDVVAGPHPERGLGKLRKLSTNVKDEGEGSKLLNLSMPSTPTTPVTPTTPTTPLSGRKADNVWRSVFHPGSNSATKTIGAQMFDKPLPNTPTVYDWLYSGETRSKHR | [5] | 115 |
| *Sesbania drummondii* | ABQ44282 | Sdr_DRM1 | MVLLEKLWDDIVAGPQPERGLGKLRKLHVKDDGEGSSKLQRNLSMPTTPTTPVTPTTPTTPVSARKVDNVWRSVFHPGSNAATKTIGAQMFDKPLPNSPTVYDWLYSGETRSKHR* |  | 115 |
| *Solanum lycopersicum* | ABH07900 | Sly_DRM1 | MVLIDKLWDDVMAGPSPDKGLGKLRKSLTIQTGGESGEGSSKYQRSLSMPASPPTPGTPVTPTNISPTVRKENVWRSVFHPGSNLATRRIGAEVFDKPSHPNAPTVYDWLYSGNTRSKHHEKC* |  | 123 |
| *Solanum tuberosum* | ABA40468 | Stu_DRM1 | MSLLDKLWDDTVAGPLPDSGLGKLRKYSTFSPRSNSGKESEVSTPRSFTEEASEDAVKVTRSIMIVKPSGSQNRDSPPVSPAGTTPPVSPFAGSAGREAFRFRRRSVSFAYENASGVGPRSPRPPYDL* |  | 128 |
| *Solanum virginianum* | AAS75891 | Svi_DRM1 | MVLIDKLWDDVMAGPSPDKGLGKLRKSLTVQTAGESSGEGSSKYQRSLSMPASPATPGTPVTPTNISPTVRKENVWRSVFHPGSNLATKRIGAEVFDKPSHPNAPTVYDWLYSGNTRSKHHEK* |  | 124 |
| *Sorghum bicolor* | XP_002460264 | Sbi_DRM1 | MGLLDQLWDDTVAGPRPDSGLGKLRKYASFSPSSSSAAAVPPSPTAEGAAAAGSGSGSGSATPAVTRSITMLRPAALSVITSPRSESSSAPSSPSPASGGAPDSPFGAATTPKGEGWKKLRRKGRMANGGDAPGTPRSPTVYDW* |  | 144 |
| *Sorghum bicolor* | XP_002465296 | Sbi_DRM2 | MGLLDKLWDDTVAGPRPDTGLGRLRKQPARPAAVKINDPAGDASAFVPPSPASGSSEETPVKVTRSIMIKRPAGYPSSPRSAASTPPASPLGTTPPISPFAAAGGRFRRKSSSDAYERATPPGTTSQPPPFEV* |  | 133 |
| *Vitis vinifera* | XP_003632757 | Vvi_DRM1 | mgfldklwdetvagpppetglgklrkykslsaarsppiinpdevqvtrsitilktnssfrnfspdsvsvpnspagssapespftpgtptgdyktdarrkaaleaferaeprsptvydwivisaldr* |  | 121 |
| *Vitis vinifera* | XP_002279836 | Vvi_DRM2 | MVLLEKLWDDVVAGPQPDRGLGKLRKLTTKPLSVKTDEGESSKYQRSMSMPASPGTPATPMTPTTPTSARKDNVWRSVFHPGSNLATKGMGSDYFDKPTKKDTPTVYDWLYSGETRTKHH* |  | 121 |
| *Vitis vinifera* | XP_002283180 | Vvi_DRM3 | MGLLDQLWDDTVAGPQPDHGLGKLRKYSTFNSRPTSGKAASDGGNGRSYSDDSSEEAMRVTRSIMIIKPPGFQNGSPPVSPAGSTPPVSPFSGGKESFRFRRRSTSDAYEKGNGVGPRSPRPPYDV* |  | 126 |
| *Zea mays* | ACG37064 | Zma_DRM1 | MGLLDQLWDETVAGPRPDSGLGKLRKYSSFSPSSSSSSSILAPAPAPAPAVTRSITIARPPSLSVESYSSSSVPSSPASTPDSPLAAATPPKADVWRRFRRKTKVSDGPEPAVGPRSPTVYDWVVISSLDR* |  | 131 |
| *Zea mays* | ACG39507 | Zma_DRM2 | MLDKLWDDVVAGPRPETGLEKLRKATTARPLVINKDADGGSYKRAQSMPSTPTTPVTPSSSSSSTTPRGAGNVWRSVFHPGSNLATKGMGANLFDRPQPNSPTVYDWLYSDETRSNHR* |  | 118 |
| *Zea mays* | NP_001130689 | Zma_DRM3 | MGLLDKLWDDTVAGPRPDTGLGRLRKQPVRPAAVKISDPAGDAAAFVPPSPASGSEETPVKVTRSIMIKRPAGYPSSPRSAASTPPASPLGTTPPISPFAGAGGRFRRKSSSDAYERATPPGTTSHPPPFDV* |  | 132 |
| *Zea mays* | NP_001150581 | Zma_DRM4 | MGLLDQLWDDTVAGPRPDSGLGKLRKYASFSPSSSAAPGSAPPPGAVTHSVTMLRRPAALSVVTSPRSESSSAPSSPSPASGAPDSPFGAATTPRGEGWKKLRGKARMGGGGGDAPGTPRSPTVYDWVVISSLDL* |  | 135 |

**References**

1 Kim HB, Lee H, Oh CJO, An CS (2007) Expression of *EuNOD-ARP1* encoding auxin-repressed protein homolog is upregulated by auxin and localized to the fixation zone in root nodules of *Elaeagnus umbellate*. Mol Cells 23: 115-121.

2 Reddy ASN, Poovaiah BW (1990) Molecular cloning and sequencing of a cDNA for an auxin-repressed mRNA: correlation between fruit growth and repression of the auxin-regulated gene. Plant Mol Biol 14: 127-136.

3 Lee SA, Gardner RC, Lay-Yee M (1993) An apple gene highly expressed in fruit. Plant Physiol 103: 1017.

4 Steiner C, Bauer J, Amrhein N, Bucher M (2003) Two novel genes are differentially expressed during early germination of the male gametophyte of *Nicotiana tabacum*. Biochim Biophys Acta 1625: 123-133.

5 Park S, Han KH (2003) An auxin-repressed gene (*RpARP*) from black locust (*Robinia pseudoacacia*) is posttranscriptionally regulated and negatively associated with shoot elongation. Tree Physiol (Oxford, U. K.) 23: 815-823.
